# GAGE-B: an evaluation of genome assemblers for bacterial organisms

**DOI:** 10.1093/bioinformatics/btt273

**Published:** 2013-05-10

**Authors:** Tanja Magoc, Stephan Pabinger, Stefan Canzar, Xinyue Liu, Qi Su, Daniela Puiu, Luke J. Tallon, Steven L. Salzberg

**Affiliations:** ^1^Center for Computational Biology, McKusick-Nathans Institute of Genetic Medicine, Johns Hopkins University School of Medicine, Baltimore, MD 21025, USA, ^2^Division for Bioinformatics, Innsbruck Medical University, 6020 Innsbruck, Austria and ^3^Institute for Genome Sciences, University of Maryland School of Medicine, Baltimore, MD 21205, USA

## Abstract

**Motivation:** A large and rapidly growing number of bacterial organisms have been sequenced by the newest sequencing technologies. Cheaper and faster sequencing technologies make it easy to generate very high coverage of bacterial genomes, but these advances mean that DNA preparation costs can exceed the cost of sequencing for small genomes. The need to contain costs often results in the creation of only a single sequencing library, which in turn introduces new challenges for genome assembly methods.

**Results:** We evaluated the ability of multiple genome assembly programs to assemble bacterial genomes from a single, deep-coverage library. For our comparison, we chose bacterial species spanning a wide range of GC content and measured the contiguity and accuracy of the resulting assemblies. We compared the assemblies produced by this very high-coverage, one-library strategy to the best assemblies created by two-library sequencing, and we found that remarkably good bacterial assemblies are possible with just one library. We also measured the effect of read length and depth of coverage on assembly quality and determined the values that provide the best results with current algorithms.

**Contact:** salzberg@jhu.edu

**Supplementary information:** Supplementary data are available at *Bioinformatics* online.

## 1 INTRODUCTION

A high-quality assembly of a bacterial genome provides the basis for research into a wide range of questions about prokaryotic biology. Increasingly in recent years, investigators have turned to rapid whole-genome sequencing of bacteria as part of efforts to trace the source of infectious disease outbreaks, to understand the source of pathogenesis and to understand multidrug resistance, among other questions. The Human Microbiome Project, which has identified thousands of new microbial strains and species as it explores the bacteria that live on our bodies, has dramatically increased the number of new bacterial genomes that are being sequenced on a daily basis. For most bacterial genome projects, the first step in analysis is the assembly of the raw ‘read’ data into larger, contiguous sequences that represent the original bacterial chromosomes.

Second- and third-generation sequencing technologies allow for remarkably fast high-throughput sequencing. The latest technologies capture longer read lengths than just a few years ago, which is expected to improve the quality of assemblies. The Illumina HiSeq machine routinely generates reads of 100 bp and can generate 600 Gb in a single run. A single lane of a HiSeq generates >35 Gb, which far exceeds what is necessary for a bacterial genome. Through multiplexing, it is feasible to generate deep sequencing data for 20–30 different bacteria in a single lane. Because the cost of preparing the DNA (including library construction) can be greater than the cost of sequencing, many researchers have begun to adopt a strategy of sequencing just a single library for each of many bacterial strains.

However, it has long been assumed that whole-genome assembly projects will include data from two or more libraries with different fragment lengths, beginning with the first bacterial genome project (Fleischmann *et al.*, 1995). A typical strategy will use one ‘short’ library, with paired reads from both ends of relatively short fragments, e.g. 200–600 bp with today’s technology. As a general rule, repeats longer than the library fragment size cannot be reliably assembled and will create gaps in the assembly. Thus, a second ‘jumping’ library will use long fragments, in the range of 2000–20 000 bp, to jump across these repeats. Next-generation assembly algorithms use these libraries to great advantage, and they are particularly important for large genomes (Schatz *et al.*, 2010).

Jumping libraries are more difficult to create, and ‘small’ fragment libraries (up to 500 bp on the HiSeq and 600 bp on the MiSeq instrument) are the fastest, most efficient way to generate deep coverage of a genome today. This motivated us to design the current study to evaluate the effectiveness of different genome assembly software on a single, short-fragment library across a range of bacterial species.

In recent years, various assembly tools have been used to assemble genomes of different sizes. Some assemblers, such as Velvet (Zerbino and Birney, 2008), were originally designed for assembling small, prokaryotic-sized genomes, whereas others, such as SOAPdenovo (Li *et al.*, 2010) and Allpaths (Gnerre *et al.*, 2011), were built to assemble large, mammalian-sized genomes. Although some assemblers might not be able to handle large genomes, almost all of them have been used in assembling bacteria. Several recent studies have compared the ability of assemblers with assemble large genomes (Barthelson *et al.*, 2011; Earl *et al.*, 2011; Gurevich *et al.*, 2013; Salzberg *et al.*, 2012; Zhang *et al.*, 2011). To date, no comprehensive evaluation has appeared that has considered which of these assemblers performs best, although hundreds (and probably thousands) of bacteria have been assembled from next-generation sequence data.

Following the standards set by the original GAGE assembly comparison (Salzberg *et al.*, 2012), GAGE-B (Genome Assembly Gold-standard Evaluation for Bacteria) evaluates how genome assemblers compare on a spectrum of bacterial genomes sequenced by the newest sequencing technologies. As with GAGE, we followed a strict standard of reproducibility, which required us to use only freely available assembly software, and our results include the full ‘recipe’ used for each assembler for each of the genomes. Our experiments were designed to answer the following questions:

- Which assembler generates the best assemblies of bacterial organisms from a single shotgun library?
- What depth of coverage and which software parameters should be used to produce the optimal assemblies?
- How does high coverage by a single library compare with the use of multiple libraries?
- How do assemblies from the longer, 250 bp MiSeq reads compare with assemblies of 100 bp HiSeq reads?

## 2 RESULTS

### 2.1 The data

We chose whole-genome shotgun data from eight bacteria, ranging in size from 2.9 to 5.4 Mb and in GC content from 33 to 69%, and for which recent Illumina sequence data are publicly available. We included several genomes for which both HiSeq and MiSeq data were available, to compare those technologies, giving us 12 datasets (Table 1). If sufficient data existed, we down-sampled reads to collect 250-fold (250×) coverage with HiSeq data and 100× coverage with MiSeq data; for a few datasets, we had to use lower coverage.

**Table 1.** Bacterial genomes and sequence read lengths used in the GAGE-B evaluation

Species	Genome size (Mb)	GC content (%)	Sequencing technology	Read length (bp)	Fragment length (bp)	Coverage
*A.hydrophila SSU*	4.7	65	HiSeq	101	180	250×
*B.cereus VD118*	5.4	35	HiSeq	101	180	100–300×
*B.cereus ATCC 10987*	5.4	35	MiSeq	250	600	100×
*B.fragilis HMW 615*	5.3	43	HiSeq	101	180	250×
*M.abscessus 6G-0125-R*	5.1	64	HiSeq	100	335	115×
*M.abscessus 6G-0125-R*	5.1	64	MiSeq	250	335^a^	100×
*R.sphaeroides 2.4.1*	4.6	69	HiSeq	101	220	210×
*R.sphaeroides 2.4.1*	4.6	69	MiSeq	251	540	100×
*S.aureus M0927*	2.9	33	HiSeq	101	180	250×
*V.cholerae CO1032(5)*	4.0	48	HiSeq	100	335	110×
*V.cholerae CO1032(5)*	4.0	48	MiSeq	250	335^a^	100×
*X.axonopodis pv. Manihotis UA323*	2.9	33	HiSeq	101	400	250×

*Note*: All datasets used paired-end reads from both ends of every fragment.

^a^The fragment lengths for two of the MiSeq libraries were relatively short, only 335 bp, because the same library was used for both HiSeq and MiSeq sequencing of those species.

The bulk of the data was downloaded from the Sequence Read Archive (SRA) at NIH’s National Center for Biotechnology Information (NCBI). The following accession numbers were used in this study: *Aeromonas hydrophila* HiSeq: SRR488186; *Bacillus cereus* HiSeq: SRR497464 and SRR497465; *Bacteroides fragilis* HiSeq: SRR488170; *Rhodobacter sphaeroides* HiSeq: SRR522244; *R.sphaeroides* MiSeq: SRR522246; *Staphylococcus aureus*-HiSeq: SRR569301; and *Xanthomonas axonopodis* HiSeq: SRR522415. The *B.cereus* MiSeq data were downloaded from the Illumina website. *Mycobacterium abscessus* and *Vibrio cholerae* were sequenced at the Institute for Genome Sciences at the University of Maryland and are available via SRA accessions SRA043447 and SRA037376, respectively, and on the GAGE-B website (ccb.jhu.edu/gage_b), as are all the reads used in this study.

Some of the bacterial genomes consist of just one chromosome (*A.hydrophila*, *B.fragilis* and *X.axonopodis*), whereas others have multiple chromosomes and/or plasmids. *V.cholerae* has two chromosomes, *B.cereus* and *M.abscessus* have one chromosome and one plasmid, *S.aureus* has one chromosome and two plasmids and *R.sphaeroides* has two chromosomes and five plasmids.

We preferentially chose species for inclusion in GAGE-B for which a finished genome was available, to allow us to compute the correctness of assemblies. The following strains were used as reference genomes: *A.hydrophila ATCC 7966* (GenBank accession number NC_008570)*, B.cereus ATCC 10987* (NC_003909, NC_005707)*, B.fragilis 638R* (NC_016776)*, M.abscessus ATCC 19977* (NC_010394, NC_010397)*, R.sphaeroides 2.4.1* (NC_007488, NC_007489m NC_007490, NC_007493, NC_007494, NC_009007, NC_009008)*, S.aureus USA300_TCH1516* (NC_010063, NC_010079, NC_012417)*, V.cholerae 01 biovar eltor str. N16961* (NC_002505, NC_002506) and *X.axonopodis pv. citrumelo* (NC_016010)*.*

For some of our sequence datasets, the finished reference sequence represents the same strain (e.g. *B.cereus ATCC 10987* and *R.sphaeroides 2.4.1*); therefore, we were able to precisely determine the accuracy of each assembly. For other species, the reference genome is a similar but distinct strain. In those cases, some differences between the assemblies and the reference genome might be true differences rather than errors, as we discuss later in the text.

For other datasets, even though the reference genome belongs to the same species, it was too divergent to use it for determining fine-grained assembly accuracy. To determine how close a reference genome was to the sequenced genome, we mapped all reads to the reference using Bowtie2 (Langmead and Salzberg, 2012) (for details, see Supplementary Material). If >90% of the reference was covered at a depth of ≥5×, we considered the two strains to be sufficiently similar. By this criterion, the reference genomes for *A.hydrophila*, *B.cereus* (HiSeq data only), *B.fragilis* and *X.axonopodis* were not similar enough to the sequenced strain, and for these assemblies, we report more limited statistics on assembly quality. Note that even if >90% of a reference genome is covered by reads, there may yet exist many true differences between the sequenced genome and the reference.

### 2.2 Data cleaning

Raw sequencing data often contain contaminants, adapter sequences or very low-quality sequences that need to be discarded. Many of the leading assemblers implement their own data cleaning steps, including adapter removal and error correction, which makes them more robust at using low-quality data. However, because some assemblers do not have a data cleaning step, and because we did not want the data quality to dominate the results, we ran a common set of data cleaning steps for all datasets. In particular, we removed adapter sequences and performed q10 quality trimming using the *ea-utils* package (Aronesty, 2011).

All datasets for each genome, both raw and trimmed, are freely available from http://ccb.jhu.edu/gage_b.

### 2.3 The assemblers

We chose eight of the leading open source genome assemblers:

- Abyss v1.3.4 (Simpson *et al.*, 2009)
- Cabog v7.0 (Miller *et al.*, 2008)
- Mira v3.4.0 (Chevreux *et al.*, 2004)
- MaSuRCA v1.8.3 (A.Zimin, manuscript under review)
- SGA v0.9.34 (Simpson and Durbin, 2012)
- SoapDenovo2 v2.04 (including GapCloser) (Luo *et al.*, 2012)
- SPAdes v2.3.0 (Bankevich *et al.*, 2012)
- Velvet v1.2.08 (Zerbino and Birney, 2008)

The one notable omission from this list is Allpaths-LG (Gnerre *et al.*, 2011), which was the best-performing assembler on large genomes in the GAGE evaluation (Salzberg *et al.*, 2012). However, Allpaths-LG explicitly requires a minimum of two libraries (a short library and a jumping library), which means it could not be run on the data used in this study.

For each genome, we ran all assemblers using different combinations of parameters on both raw and cleaned data. To select the best genome assembly for each genome and for each software tool, we chose the one that produced the largest N50 contig size, which is a common heuristic for selecting the best assembly when the true genome sequence is unknown.

**Fig. 1.**
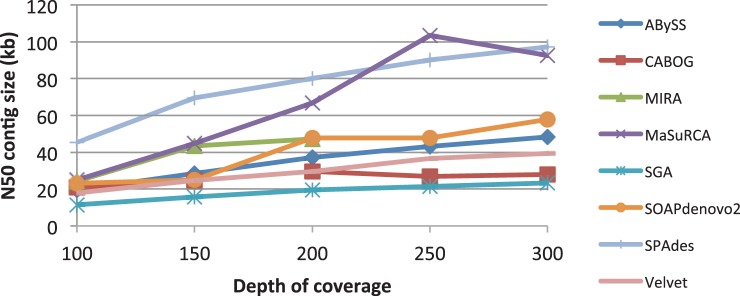
Comparison of N50 contig size (in kilobases) on the *y*-axis, versus depth of coverage on the *x*-axis, for the eight assemblers used in this study. All datasets were 100 bp HiSeq reads from *B.cereus*

*Depth of coverage adjustments.* Before assembling the genomes, we ran preliminary experiments on one dataset to optimize the depth of coverage. We used 100 bp reads from the *B.cereus* data, which yielded ∼300× coverage, to experiment with different depths. We randomly down-sampled these reads to produce coverages of 250, 200, 150 and 100×. We then assembled the genome at each coverage depth, by each assembler, using multiple combinations of parameters. In Figure 1, we show the N50 contig sizes for the best assembly from each assembler at each coverage depth. As the figure shows, contig size steadily increases from 100 to 250×. Above 250×, contig size increased more slowly for some assemblers and decreased or held steady for others; therefore, we used 250× coverage with 100 bp reads for all genomes where sufficient data were available.

### 2.4 The assemblies

In Tables 2 and 3, Supplementary Tables S1–S12 and Supplementary Figures S1–S2, we present various statistics on the performance of each assembler on all 12 datasets. We used the following metrics for both contigs and scaffolds:

- The number of contigs (or scaffolds) at least 200 bp long (500 bp for scaffolds).
- N50 size, which is the size of the smallest contig such that 50% of the genome is contained in contigs of size N50 or larger. For example, for a 5.0 Mb genome, the contig N50 size would be computed by adding up the contig sizes from largest to smallest until the cumulative size was >2.5 Mb. The size of the smallest contig in this set is the N50 size.
- Nx statistics (Supplementary Figs S1 and S2) are defined similarly to N50, where the Nx size is the length of the smallest contig such that x% of the genome is contained in the contigs of size Nx or larger.
- Errors, determined by comparison with the reference genome. We defined this as the sum of the number of relocations, translocations and inversions affecting at least 1000 bp. A relocation is defined as a misjoin in a contig/scaffold such that if the contig/scaffold is split into two pieces at the misjoin, then the left and right pieces map to distinct locations on the reference genome that are separated by at least 1000 bp, or that overlap by at least 1000 bp. A translocation is defined as a misjoin where the left and the right pieces map to different chromosomes or plasmids. An inversion is defined as a misjoin such that the left and the right pieces map to opposite strands on the same chromosome.
- Local errors, defined as misjoins where the left and right pieces map onto the reference genome to distinct locations that are <1000 bp apart, or that overlap by <1000 bp.
- Corrected N50 size, defined as the N50 size obtained after splitting contigs/scaffolds at each error. Note that local errors were not used for the purpose of calculating corrected N50 values.
- The fraction of the reference genome covered by contigs/scaffolds.
- The number of unaligned contigs, computed as the number of contigs that MUMmer (Delcher *et al.*, 1999, 2002; Kurtz *et al.*, 2004) was not able to align, even partially, to the reference genome.
- Duplication ratio, an approximation of the amount of overlaps among contigs/scaffolds that should have been merged. Failure to merge overlaps leads to overestimation of the genome size and creates two copies of sequences that exist in just one copy.
- The number of proteins fully contained in contigs. This was computed by aligning all annotated proteins from the reference genome using tblastn (Altschul *et al.*, 1997), which translates the reference genome in all six frames. These alignments can be detected even when the DNA sequence is too divergent to align easily. This metric provides an alternative measure of assembly completeness and accuracy when the closest finished genome is too divergent. Supplementary Figure S3 shows that the percent identity of proteins across strains can range well below 80%.

**Table 2.** Comparison of corrected N50 contig sizes, shown in kilobases, for assemblies where the finished reference genome was identical or near-identical

Assembler	Species assembled						
	HiSeq (100 bp) reads			MiSeq (250 bp) reads			
	*R*.*sphaeroides*	*M*.*abscessus*	*V*.*cholerae*	*B*.*cereus*	*R*.*sphaeroides*	*M*.*abscessus*	*V*.*cholerae*
ABySS	13.0	115.7	93.0	130.6	21.4	68.5	60.3
CABOG	11.2	78.2	48.8	150.5	30.5	8.3	32.5
MIRA	17.7	129.2	87.1	100.0	15.4	75.0	108.7
MaSuRCA	**176.8**	**194.0**	**236.4**	**246.7**	**130.7**	36.2	71.6
SGA	12.1	27.9	23.4	25.5	9.1	12.8	27.3
SOAPdenovo	10.5	147.2	106.5	**246.3**	33.5	113.3	65.5
SPAdes	83.5	147.9	77.1	103.7	118.1	**215.4**	**246.6**
Velvet	13.1	60.3	39.5	24.5	24.2	41.5	67.1

*Note*: The best values (or two best, in case of near-ties) for each genome are shown in bold. Corrected N50 is defined in Section 2.4.

**Table 3.** Comparison of N50 sizes (in kilobases) for assemblies where the sequenced strain was too divergent to compute a corrected N50 value

Assembler	Species assembled				
	*A.hydrophila*	*B*.*cereus*	*B*.*fragilis*	*S*.*aureus*	*X*.*axonopodis*
ABySS	237.5	48.3	146.2	73.9	89.9
CABOG	278.4	61.6	94.2	102.8	105.8
MIRA	246.2	47.4	134.3	132.4	105.6
MaSuRCA	**828.6**	**103.6**	**158.7**	**221.8**	**117.9**
SGA	68.8	23.4	41.2	38.1	47.8
SOAPdenovo	243.9	57.9	116.1	146.3	74.2
SPAdes	379.7	97.2	**157.7**	187.1	**117.5**
Velvet	184.4	38.9	125.2	122.5	83.0

*Note*: Boldface indicates the best result in each column, with the top two results highlighted when the difference was minimal. All genomes shown here were assembled from 100 bp HiSeq reads. See the Supplementary data for additional statistics.

With the exception of the number of proteins contained in contigs, we calculated all metrics using the QUality ASsesment Tool for genome assembly (Gurevich *et al.*, 2013).

### 2.5 Comparison of assemblies

For seven of our genome datasets, the raw sequence data derived from a strain were either identical or nearly identical to the finished genome. This allowed us to compute a precise value for the corrected N50 sizes. Whenever an assembler incorrectly merges two contigs, the resulting assembly will seem to have a bigger N50 size, thus creating an inferior assembly with an apparently ‘better’ N50 size. By breaking contigs at misjoins, the corrected N50 provides a better measure of assembly quality.

Three of the sequence datasets precisely matched the reference genome: *R.sphaeroides* (two datasets, MiSeq and HiSeq) and *B.cereus* (MiSeq data only). Two other genomes, *M.abscessus* and *V.cholerae*, came from near-identical strains, and these include both MiSeq and HiSeq data. We summarize the corrected N50 contig sizes for all seven of these datasets in Table 2. For the near-identical strains, some disagreements between sequenced genomes and the references might actually be true differences between strains. In such cases, we expect that all assemblies will disagree with the reference genome, and thus the comparisons between assemblies will still be valid, although the number of reported errors might be slightly inflated.

Although all genomes were chosen for GAGE-B based on the existence of a reference genome, our analyses showed that for five datasets, the sequenced genome represented a strain that, although belonging to the same species, had an unexpected amount of divergence from the reference strain. This made it difficult to distinguish assembly errors from true differences, which made it impossible to report a trustworthy corrected N50 size. We report the uncorrected N50 contig size for all assemblies of these five genomes in Table 3.

Much more detail on each genome is contained in Supplementary Tables S1–S12. Here, we highlight some of the key results from these tables.

In the MiSeq assembly of *B.cereus* (Supplementary Table S1), MaSuRCA and SOAPdenovo generated contigs with the highest N50 and corrected N50 values. These two assemblies produced N50 values that are more than twice the size of any other assembly, and more than five times bigger than the worst assemblies. SOAPdenovo and ABySS made the fewest errors; however, they also produced the highest number of local errors. SOAPdenovo generated the largest scaffolds, but again had more local errors than most other assemblies. All assemblies covered >99% of the genome by both contigs and scaffolds. SPAdes generated a large number of small, unaligned contigs (36 941), of which nearly 100% had coverage of ≥3×, indicating that they should be discarded.

For the HiSeq assemblies of *R.sphaeroides* (Supplementary Table S2), MaSuRCA had the highest contig N50 values at 176.8 kb, followed by SPAdes at 83.5 kb. All of the other assemblies were far more fragmented, with N50 sizes ranging from 10.1 to 17.7 kb. Thus, for this genome, the choice of assembler seems to have a large impact on the quality of the resulting assembly. Looking at the MiSeq data for the same genome (Supplementary Table S3), the results are similar: MaSuRCA and SPAdes produced large contigs, and most other assemblers had N50 values four to five times smaller. The results for scaffolds showed the same relative performance. Looking at the number of proteins fully contained within contigs, SPAdes emerged as a clear winner with 3562 compared with the next closest competitor at 3369.

For the *M.* *abscessus* assemblies (Supplementary Tables S4 and S5), MaSuRCA had the largest contig N50 size at 246.9 kb. The other assemblers—MIRA, SOAPdenovo and SPAdes—had N50 sizes of ∼150 kb for the HiSeq data. Noteworthy here is that although most assemblers performed worse with the MiSeq data, SPAdes performed considerably better, with an N50 contig size of 215.4 kb. The MIRA assembly, despite its large N50 contig size, had many more errors than any other assembler on this species, although a high-error rate was not observed on other genomes. Despite these errors, its corrected contig N50 was large, suggesting that the errors occur in smaller contigs.

The largest contigs for *V.cholerae* (Supplementary Tables S6 and S7) were produced by SPAdes from 250 bp reads. MaSuRCA produced contigs that were nearly as large from the 100 bp data. Both assemblers produced contig N50 sizes >225 kb, whereas most other assemblers were <100 kb. SOAPdenovo and MIRA produced contig N50 sizes close to 100 kb, but with a larger number of errors than most other methods.

The *A.hydrophila* assembly (Supplementary Table S8) is particularly worth noting because of the remarkably large N50 size that MaSuRCA produced, 828.6 kb. The assembly contained only 32 contigs for this 4.75 Mb genome, the smallest number for any of our experiments. Most of the other assemblers also produced large contigs for this genome, indicating that it was the ‘easiest’ to assemble of all the GAGE-B datasets. For genomes such as this one, sequencing with just a single library is clearly a good strategy.

At the other end of the spectrum, the *B.cereus* VD118 strain, which was only available in 100 bp reads, presented the greatest difficulties for all of the assemblers. The best contig N50 sizes were just above and below 10 kb, and no assembly had fewer than 164 contigs. The other *B.cereus* strain yielded better assemblies, but because this was based on 250 bp reads, it is hard to ascertain the precise reason for the differences.

### 2.6 Combination of assemblies

We also investigated whether combining two different genome assemblies can improve the overall assembly quality. We combined the output of different algorithms, as well as the same algorithm run with varying k-mer sizes. Intuitively, the latter aims at capturing the benefits of a large value of k, namely, fewer false overlaps, and a small value of k, namely, fewer missed overlaps. Note that SPAdes already incorporates this strategy into its algorithm by using a multi-kmer de Bruijn graph.

We evaluated the performance of post-assembly merging by two different tools, minimus2 (Sommer *et al.*, 2007) from the AMOS package and the Graph Accordance Assembly (GAA) system (Yao *et al.*, 2012). The minimus2 pipeline uses nucmer (Kurtz *et al.*, 2004) to compute overlaps between contigs and returns co-assembled contigs and unmerged singletons separately. Following the scheme introduced in Howe *et al.* (2012), we used CD-HIT (Fu *et al.*, 2012) to remove contigs that were 99% similar before merging them. GAA treats one assembly as the query, which it aligns to the other, called the target. It represents overlap information from these alignments in a graph, and then finds maximal paths in the graph to produced merged contigs. The MAIA algorithm (Nijkamp *et al.*, 2010) follows a similar strategy for merging multiple input assemblies, but MAIA requires a reference genome and thus was not considered in our comparison.

We ran these experiments on the *B.cereus* MiSeq dataset because we had a finished reference from a near-identical strain. We focused on combining contigs rather than scaffolds because no jumping library was available. Over a large number of computational experiments, most combinations of assemblers, k-mer values and merging algorithms did not produce improvements, and they often produced inferior assemblies to the best individual assembly (see Supplementary Material and Supplementary Tables S15–S20).

However, one merging strategy did yield some improvements. When using GAA for contig merging with the SOAPdenovo assembly as the target, the assembly was improved substantially when using ABySS, CABOG or MaSuRCA as the query assembly (Supplementary Table S21). In all three cases, the corrected contig N50 size improved to 458 kb, an increase of >80% compared with the best individual assembly (∼246 kb, by both MaSuRCA and SOAPdenovo). The total number of contigs was unchanged, at ∼90. Another noteworthy improvement using this strategy was for the CABOG assembly, using the ABySS assembly as the query, for which the corrected N50 size improved from 151 kb to 288 bp (a 90% increase).

Based on these results, it seems that an alignment-based approach to merge assemblies from different algorithms can indeed yield an improvement compared with single high-quality assemblies. However, finding a pair of algorithms that complement each other in an advantageous manner may require extensive trial and error experiments.

## 3 DISCUSSION

The statistics presented in Tables 2 and 3 and Supplementary Tables S1–S12 support a number of conclusions about the capabilities of the various assembly methods and provide answers to the four questions posed in Section 1.

First, we consider which assembler generates the best assemblies of bacterial species from a single whole-genome shotgun library. Although no assembler won on all the various metrics, the MaSuRCA assembler had the largest contig sizes, measured by either N50 or corrected N50 values, for 10 of the 12 experiments. The SPAdes assembler, a relatively recent entry into the next-generation assembly field, came in first or essentially tied for first for 4 of the 12 genomes. These results were consistent across both 100 (HiSeq) and 250 bp (MiSeq) reads, although SPAdes had a larger boost in improvement for the longer reads.

When considering the number of errors, including local errors, ABySS and SGA consistently produced assemblies with the fewest errors. These assemblers also tended to produce smaller contigs than most of the others, suggesting that they use a conservative assembly strategy that trades off contig size for accuracy. This result is consistent with the results of the original GAGE evaluation (Salzberg *et al.*, 2012) on larger genomes.

In most cases, the number of errors did not greatly reduce the N50 sizes, because of, at least in part, the distinction made here between ‘local’ errors, which involve insertions, deletions and rearrangements <1 kb and larger errors. We only split contigs and scaffolds on larger errors for this study, in contrast to our earlier GAGE study where we split on all errors >5 bp. We noted that for some assemblers, the number of local errors tended to be larger, e.g. SOAPdenovo had 50 local errors in its assembly of *V.cholerae* from 100 bp reads, whereas the other methods had only 0–18 local errors. These errors would further reduce N50 values if they were used to split assemblies.

A new quality metric that we computed across all 12 datasets (Supplementary Tables S1–S12) was the number of proteins contained fully within contigs. This metric is tolerant of divergence between the sequenced strain and the reference because protein sequences diverge much more slowly than nucleotide sequences. In addition, this metric captures a feature of biological interest: whether a typical protein is fully contained within a contig. For this computation, we used all annotated protein coding genes from the reference genome. On this metric, the SPAdes assemblies performed the best, with the largest value for 10 of the 12 genomes. For most genomes, multiple assemblers performed similarly, and there was no clear winner.

Overall, MaSuRCA and SPAdes produced the best assemblies across these 12 bacterial organisms. However, even these assemblers have some weaknesses that should be pointed out. SPAdes sometimes generated many small contigs that did not align to the reference genome. On inspection, we found that most of these had low coverage, and because SPAdes provides detailed coverage information, one can easily filter out the low-coverage contigs. MaSuRCA has a different problem: it sometimes creates good contigs that it labels ‘degenerate’ based on internal coverage statistics, which can cause it to omit some parts of the genome from the assembly. This problem can be solved simply by including degenerate contigs above some minimum length threshold as part of the assembly.

One of our primary motivations in GAGE-B was to answer the question of whether deep sequencing from a single, short-fragment library can produce an assembly that is comparable with what we get from two libraries, especially when one is a jumping library. One answer to this question can be found by comparing the best assemblies of *R.sphaeroides* (by MaSuRCA and SPAdes) with the best assembly (by Allpaths-LG) from the original GAGE study, which used two libraries. For comparison, we also ran MaSuRCA on the two-library dataset, although we did not run SPAdes because it is not yet designed to handle two libraries.

As shown in Table 4, the contigs created by both MaSuRCA and SPAdes from a single deep-coverage library were considerably larger than those from the two-library data, which was at lower coverage (100×). The number of errors was also slightly lower, although this could be due to improvements in assembly algorithms. However, the lack of long ‘jumping’ pairs makes significant difference in the size of scaffolds. A single library of paired reads from relatively short fragments simply cannot span many of the repetitive sequences in a genome. Thus, although the biggest scaffold with the two-library strategy was over 2.5 Mb in length and spanned more than half of the main chromosome, the best scaffolds for the one-library assembly were less than one-tenth as long. We conclude that for gene-level questions, a single-sequencing library today, made from either 100 or 250 bp reads, will produce a good *de novo* assembly in which over half the genome is contained in contigs >100 kb, and in which a large majority of genes are contained within scaffolds. If a closely related genome is available, it can be used to aid scaffolding, but otherwise a jumping library is still necessary to produce truly large scaffolds.

**Table 4.** Assemblies of *R.sphaeroides* using one versus two libraries

Assembler (dataset)	MaSuRCA (one HiSeq library)	MaSuRCA (GAGE setting: two libraries)	SPAdes (one MiSeq library)	Allpaths-LG (GAGE setting: two libraries)
Contigs				
Number	**130**	258	185	204
Errors	**5**	11	9	15
Local errors	**5**	7	**5**	12
Corrected N50 (kb)	**177**	30	118	43
Scaffolds				
Number	101	**25**	73	33
Errors	**4**	9	12	15
Local errors	**8**	71	9	81
Corrected N50 (kb)	197	2528	152	**2925**

*Note*: Shown are results from MaSuRCA and SPAdes, the two assemblers with the best performance in this study on a single library, and from Allpaths-LG, which was the best performer on the two-library dataset in the original GAGE study. We also include an additional comparison using MaSuRCA on two libraries. The best values in each row are shown in boldface type.

We also considered the question of whether 250 bp reads are superior to 100 bp reads at a comparable cost. Because these reads are more expensive to produce, we used 2.5-fold lower coverage (100 versus 250×) for the long reads. We obtained both read lengths for three species: *M.abscessus, R.sphaeroides* and *V.cholerae*, for all of which we had a near-identical finished genome. Tables 2 and 3 and Supplementary Tables S2–S8 show the details of these assemblies.

For *M.abscessus* and *V.cholerae*, the best assemblies for the 100 and 250 bp reads had similar N50 sizes, although for *R.sphaeroides*, the best N50 was 30% larger with 100 bp reads. The number of proteins contained in contigs was higher in all 100 bp assemblies except for those built by SPAdes. It may be tempting to conclude that 100× coverage in MiSeq reads is inferior to 250× coverage in HiSeq reads, but in looking at the details, we observed that among all the assemblies of *R.sphaeroides*, five had higher contig N50 values when based on MiSeq versus HiSeq data. For *V.cholerae*, four assemblers performed better with MiSeq data and the other four performed better with HiSeq data. We also observed that MaSuRCA always generated better results using HiSeq datasets, whereas SPAdes always generated better results using MiSeq data. Other assemblers did not show such an obvious preference. Our hypothesis is that many algorithms have not yet had time to adapt to the longer 250 bp reads, and once they do, lower coverage with longer reads may be superior, as it already is in some cases.

Overall, our results support a conclusion that with deep sequence coverage, the latest genome assemblers can produce good *de novo* assemblies from just a single, short-fragment DNA library. Today this strategy represents the lowest-cost method for capturing the entire genome of a bacterium or other species with small genomes. The vast majority of protein-coding genes will be contained wholly within contigs using this strategy, although an important caveat is that large-scale changes in genome structure, particularly large rearrangements, will likely not be captured. Our findings suggest that multiplexing many genomes in the same sequencing run will provide a highly effective means for studying hundreds if not thousands of bacterial strains in the near future.

## Supplementary Material

Supplementary Data

## References

[btt273-B1] Altschul SF (1997). Gapped BLAST and PSI-BLAST: a new generation of protein database search programs. Nucleic Acids Res..

[btt273-B2] Aronesty E (2011). Ea-utils: command-line tools for processing biological sequencing data.

[btt273-B3] Bankevich A (2012). SPAdes: a new genome assembly algorithm and its applications to single-cell sequencing. J. Comput. Biol..

[btt273-B4] Barthelson R (2011). Plantagora: modeling whole genome sequencing and assembly of plant genomes. PLoS One.

[btt273-B5] Chevreux B (2004). Using the miraEST assembler for reliable and automated mRNA transcript assembly and SNP detection in sequenced ESTs. Genome Res..

[btt273-B6] Delcher AL (1999). Alignment of whole genomes. Nucleic Acids Res..

[btt273-B7] Delcher AL (2002). Fast algorithms for large-scale genome alignment and comparison. Nucleic Acids Res..

[btt273-B8] Earl D (2011). Assemblathon 1: a competitive assessment of de novo short read assembly methods. Genome Res..

[btt273-B9] Fleischmann RD (1995). Whole-genome random sequencing and assembly of *Haemophilus influenzae* Rd. Science.

[btt273-B10] Fu L (2012). CD-HIT: accelerated for clustering the next-generation sequencing data. Bioinformatics.

[btt273-B11] Gnerre S (2011). High-quality draft assemblies of mammalian genomes from massively parallel sequence data. Proc. Natl Acad. Sci. USA.

[btt273-B12] Gurevich A (2013). QUAST: quality assessment tool for genome assemblies. Bioinformatics.

[btt273-B13] Howe AC (2012). Assembling large, complex environmental metagenomes. arXiv.

[btt273-B14] Kurtz S (2004). Versatile and open software for comparing large genomes. Genome Biol..

[btt273-B15] Langmead B, Salzberg SL (2012). Fast gapped-read alignment with Bowtie 2. Nat. Methods.

[btt273-B16] Li R (2010). *De novo* assembly of human genomes with massively parallel short read sequencing. Genome Res..

[btt273-B27] Luo R (2012). SOAPdenovo2: an empirically improved memory-efficient short-read de novo assembler. GigaScience.

[btt273-B17] Miller JR (2008). Aggressive assembly of pyrosequencing reads with mates. Bioinformatics.

[btt273-B18] Nijkamp J (2010). Integrating genome assemblies with MAIA. Bioinformatics.

[btt273-B19] Salzberg SL (2012). GAGE: a critical evaluation of genome assemblies and assembly algorithms. Genome Res..

[btt273-B20] Schatz MC (2010). Assembly of large genomes using second-generation sequencing. Genome Res..

[btt273-B21] Simpson JT, Durbin R (2012). Efficient *de novo* assembly of large genomes using compressed data structures. Genome Res..

[btt273-B22] Simpson JT (2009). ABySS: a parallel assembler for short read sequence data. Genome Res..

[btt273-B23] Sommer DD (2007). Minimus: a fast, lightweight genome assembler. BMC Bioinformatics.

[btt273-B24] Yao G (2012). Graph accordance of next-generation sequence assemblies. Bioinformatics.

[btt273-B25] Zerbino DR, Birney E (2008). Velvet: algorithms for de novo short read assembly using de Bruijn graphs. Genome Res..

[btt273-B26] Zhang W (2011). A practical comparison of *de novo* genome assembly software tools for next-generation sequencing technologies. PLoS One.

