# Supplementary material for: GAGE-B: an evaluation of genome assemblers for bacterial organisms
Source: Bioinformatics. 2013 May 10;29(14):1718–25. doi: 10.1093/bioinformatics/btt273 (PMC3702249; doi:10.1093/bioinformatics/btt273)
Supplement: Supplementary Data [file supp_29_14_1718__index.html]

GAGE-B: an evaluation of genome assemblers for bacterial organisms — GAGE-B: an evaluation of genome assemblers for bacterial organisms — Supplementary Data 

# GAGE-B: an evaluation of genome assemblers for bacterial organisms

## Supplementary Data

files

**Files in this Data Supplement:**

- Supplementary Data - docx file
