# Supplementary material for: GAGE-B: an evaluation of genome assemblers for bacterial organisms
Source: Bioinformatics. 2013 May 10;29(14):1718–25. doi: 10.1093/bioinformatics/btt273 (PMC3702249; doi:10.1093/bioinformatics/btt273)
Supplement: Supplementary Data [file supp_btt273_GAGE-B_SupplementaryMaterial_Apr4.docx]

**GAGE–B: An Evaluation of Genome Assemblers for Bacterial Organisms: Supplementary Material**

**Supplementary Tables for assemblies of all twelve genome data sets**

The following tables contain detailed assembly statistics for all assembly programs compared in GAGE-B, showing both contig and scaffold statistics. Greater detail is provided for the seven genomes that are identical or near-identical to the reference, as described in the main text. For the five more-divergent genomes, we report only the N50 statistics and the number of proteins (taken from the annotation of the reference genome) fully contained within contigs.

**Supplementary Table S1.** Assemblies of *B. cereus* from 250bp MiSeq reads. The reference genome size is 5,432,652bp and its annotation contains 5,843 proteins. The best value in each row is shown in boldface. N50, Errors, and Duplication are defined in the main text. GenFrac: the percentage of the reference genome covered by the assembly. Unaligned: the number of contigs that could not be aligned to the reference genome. Proteins: the number of proteins from the reference genome that are fully contained within contigs. Complete assembly recipes, including parameters used for each assembler, are available below.

|  |  | ABySS | CABOG | MIRA | MaSuRCA | SGA | SOAPdenovo | SPAdes | Velvet |
| --- | --- | --- | --- | --- | --- | --- | --- | --- | --- |
| Contigs | Num | 115 | **78** | 153 | 90 | 3335 | 105 | 49967 | 404 |
|  | N50 (kb) | 130.6 | 155.4 | 116.5 | **246.7** | 25.5 | 246.3 | 103.7 | 24.5 |
|  | Errors | 3 | 8 | 11 | 15 | 26 | **1** | 21 | 5 |
|  | Errors-L | 27 | **6** | 18 | 10 | **6** | 22 | 15 | 13 |
|  | N50Corr (kb) | 130.6 | 150.5 | 100.0 | **246.7** | 25.5 | 246.3 | 103.7 | 24.5 |
|  | GenFrac (%) | 99.3 | 99.5 | **99.98** | 99.8 | 99.96 | 98.96 | **99.98** | 99.1 |
|  | Unaligned | 2 | **0** | 7 | **0** | 10 | **0** | 36941 | **0** |
|  | Duplication | **1.0** | **1.0** | **1.0** | **1.0** | 2.2 | **1.0** | 1.6 | **1.0** |
|  | Proteins | 5346 | 5413 | 5397 | **5439** | 5266 | 5410 | 5410 | 5152 |
| Scaffolds | Num | 74 | **33** | n/a | 61 | 341 | 56 | 73 | 78 |
|  | N50 (kb) | 135.6 | 431.5 | n/a | 337.9 | 25.5 | **456.6** | 212.5 | 247.7 |
|  | Errors | 4 | 12 | n/a | 17 | 4 | **1** | 11 | 9 |
|  | Errors-L | 31 | 17 | n/a | 13 | **1** | 43 | 37 | 268 |
|  | N50Corr(kb) | 135.3 | 348.3 | n/a | 337.9 | 25.5 | **456.0** | 212.5 | 208.4 |
|  | GenFrac (%) | 99.1 | 99.5 | n/a | 99.7 | 97.7 | 98.8 | **99.96** | 99.1 |
|  | Unaligned | **0** | **0** | n/a | **0** | **0** | **0** | 2 | **0** |
|  | Duplication | **1.0** | **1.0** | n/a | **1.0** | **1.0** | **1.0** | **1.0** | **1.0** |

**Supplementary Table S2**. Assemblies of *R. sphaeroides* HiSeq data (reference genome size: 4,565,960bp, number of proteins: 4,242). Definitions of the various metrics are the same as in Table S1.

|  |  | ABySS | CABOG | MIRA | MaSuRCA | SGA | SOAPdenovo | SPAdes | Velvet |
| --- | --- | --- | --- | --- | --- | --- | --- | --- | --- |
| Contigs | Num | 603 | 537 | 1224 | **130** | 838 | 859 | 298 | 696 |
|  | N50 (kb) | 13.3 | 11.4 | 17.7 | **176.8** | 12.1 | 10.1 | 83.5 | 13.1 |
|  | Errors | 9 | 4 | 12 | 5 | **2** | 3 | 8 | 3 |
|  | Errors-L | **2** | 3 | 7 | 5 | **2** | 12 | 5 | 5 |
|  | N50Corr(kb) | 13.0 | 11.2 | 17.7 | **176.8** | 12.1 | 10.5 | 83.5 | 13.1 |
|  | GenFrac (%) | 98.9 | 91.3 | **99.9** | 97.7 | 93.2 | 97.6 | **99.9** | 98.6 |
|  | Unaligned | 1 | **0** | **0** | **0** | **0** | 1 | 107 | 1 |
|  | Duplication | **1.0** | **1.0** | **1.0** | **1.0** | **1.0** | **1.0** | **1.0** | **1.0** |
|  | Proteins | 3269 | 2974 | 3189 | 3489 | 2997 | 3027 | **3521** | 3183 |
| Scaffolds | Num | 518 | 320 | n/a | **101** | 481 | 555 | 129 | 283 |
|  | N50 (kb) | 13.5 | 21.2 | n/a | **196.5** | 11.8 | 15.5 | 127.9 | 34.2 |
|  | Errors | 27 | 5 | n/a | 4 | **1** | 3 | 8 | 12 |
|  | Errors-L | 13 | 32 | n/a | 8 | **2** | 135 | 9 | 339 |
|  | N50Corr (kb) | 13.3 | 20.6 | n/a | **196.5** | 11.8 | 15.5 | 127.9 | 33.1 |
|  | GenFrac (%) | 98.5 | 91.3 | n/a | 97.7 | 86.3 | 96.6 | **99.8** | 98.1 |
|  | Unaligned | **0** | **0** | n/a | **0** | **0** | 7 | **0** | **0** |
|  | Duplication | **1.0** | **1.0** | n/a | **1.0** | **1.0** | **1.0** | **1.0** | **1.0** |

**Supplementary Table S3**. Assemblies of *R. sphaeroides* MiSeq data (reference genome size: 4,565,960bp, number of proteins: 4,242)

|  |  | ABySS | CABOG | MIRA | MaSuRCA | SGA | SOAPdenovo | SPAdes | Velvet |
| --- | --- | --- | --- | --- | --- | --- | --- | --- | --- |
| Contigs | Num | 485 | 146 | 867 | **63** | 986 | 437 | 185 | 415 |
|  | N50 (kb) | 21.4 | 31.5 | 15.8 | **130.7** | 9.1 | 33.5 | 118.1 | 24.0 |
|  | Errors | **2** | 6 | 18 | 4 | 5 | 4 | 9 | 3 |
|  | Errors-L | **3** | **3** | 7 | 6 | **3** | 14 | 5 | 10 |
|  | N50Corr(kb) | 21.4 | 30.5 | 15.4 | **130.7** | 9.1 | 33.5 | 118.1 | 24.2 |
|  | GenFrac (%) | 99.3 | 85.8 | 99.8 | 92.3 | 99.4 | 98.8 | **99.9** | 98.7 |
|  | Unaligned | **0** | **0** | **0** | 1 | 3 | 19 | 54 | 1 |
|  | Duplication | **1.0** | **1.0** | **1.0** | **1.0** | **1.0** | **1.0** | **1.0** | **1.0** |
|  | Proteins | 3362 | 3052 | 3208 | 3314 | 3104 | 3353 | **3562** | 3369 |
| Scaffolds | Num | 382 | 131 | n/a | **52** | 733 | 185 | 73 | 143 |
|  | N50 (kb) | 21.4 | 40.3 | n/a | 144.8 | 8.0 | 45.1 | **151.8** | 85.3 |
|  | Errors | 2 | 6 | n/a | 3 | **0** | 5 | 12 | 14 |
|  | Errors-L | 3 | 7 | n/a | 10 | **2** | 220 | 9 | 192 |
|  | N50Corr (kb) | 21.4 | 36.1 | n/a | 144.8 | 8.0 | 45.0 | **151.8** | 82.5 |
|  | GenFrac (%) | 98.6 | 86.8 | n/a | 92.3 | 88.3 | 98.6 | **99.9** | 98.2 |
|  | Unaligned | **0** | **0** | n/a | **0** | **0** | **0** | **0** | **0** |
|  | Duplication | **1.0** | **1.0** | n/a | **1.0** | **1.0** | **1.0** | **1.0** | **1.0** |

**Supplementary Table S4**. Assemblies of *M. abscessus* HiSeq data (reference genome size: 5,090,401bp, number of proteins: 4,941)

|  |  | ABySS | CABOG | MIRA | MaSuRCA | SGA | SOAPdenovo | SPAdes | Velvet |
| --- | --- | --- | --- | --- | --- | --- | --- | --- | --- |
| Contigs | Num | 124 | 127 | 3241 | **66** | 377 | 91 | 96 | 155 |
|  | N50 (kb) | 119.4 | 81.4 | 147.3 | **246.9** | 28.8 | 148.6 | 150.3 | 61.6 |
|  | Errors | 4 | 7 | 598 | 4 | **3** | 7 | **3** | **3** |
|  | Errors-L | 12 | 8 | 8 | 5 | **4** | 23 | 8 | 12 |
|  | N50Corr (kb) | 115.7 | 78.2 | 129.2 | **194.0** | 27.9 | 147.2 | 147.9 | 60.3 |
|  | GenFrac (%) | 99.3 | 98.9 | **99.4** | **99.4** | 99.3 | **99.4** | **99.4** | 99.3 |
|  | Unaligned | **0** | **0** | 58 | **0** | **0** | 3 | 7 | 1 |
|  | Duplication | **1.0** | **1.0** | 1.2 | **1.0** | **1.0** | **1.0** | **1.0** | **1.0** |
|  | Proteins | 4308 | 4112 | **4350** | 4298 | 4200 | 4348 | **4350** | 4286 |
| Scaffolds | Num | 64 | 109 | n/a | **56** | 301 | 66 | 57 | 59 |
|  | N50 (kb) | 147.9 | 94.4 | n/a | 246.8 | 28.8 | 150.3 | 223.1 | **262.0** |
|  | Errors | 8 | 10 | n/a | 9 | **3** | 7 | 6 | 6 |
|  | Errors-L | 33 | 15 | n/a | 7 | **4** | 29 | 11 | 87 |
|  | N50Corr (kb) | 127.4 | 90.9 | n/a | **194.0** | 27.9 | 147.9 | 213.4 | 148.7 |
|  | GenFrac (%) | 99.2 | 98.9 | n/a | **99.4** | 98.9 | 99.3 | **99.4** | 99.2 |
|  | Unaligned | **0** | **0** | n/a | **0** | **0** | 1 | **0** | **0** |
|  | Duplication | **1.0** | **1.0** | n/a | **1.0** | **1.0** | **1.0** | 1.1 | **1.0** |

**Supplementary Table S5**. Assemblies of *M. abscessus* MiSeq data (reference genome size: 5,090,491bp, number of proteins: 4,941)

|  |  | ABySS | CABOG | MIRA | MaSuRCA | SGA | SOAPdenovo | SPAdes | Velvet |
| --- | --- | --- | --- | --- | --- | --- | --- | --- | --- |
| Contigs | Num | 209 | 857 | 1751 | 326 | 1114 | **113** | 908 | 279 |
|  | N50 (kb) | 70.4 | 8.7 | 114.1 | 38.2 | 13.3 | 131.6 | **220.2** | 48.2 |
|  | Errors | **3** | 123 | 2367 | 70 | 178 | 4 | 21 | 78 |
|  | Errors-L | 4 | 6 | 43 | **3** | 5 | 27 | 8 | 5 |
|  | N50Corr (kb) | 68.5 | 8.3 | 75.0 | 36.2 | 12.8 | 113.3 | **215.4** | 41.5 |
|  | GenFrac (%) | 99.3 | 96.2 | **99.4** | 98.4 | **99.4** | 99.3 | **99.4** | 99.1 |
|  | Unaligned | 10 | 5 | 76 | **1** | 8 | 2 | 810 | 52 |
|  | Duplication | **1.0** | **1.0** | 1.2 | 1.1 | **1.0** | **1.0** | **1.0** | **1.0** |
|  | Proteins | 4297 | 3688 | 4323 | 4254 | 3974 | 4333 | **4361** | 4223 |
| Scaffolds | Num | 147 | 847 | n/a | 324 | 664 | 79 | **73** | 154 |
|  | N50 (kb) | 73.2 | 9.1 | n/a | 38.2 | 13.3 | 152.6 | **220.2** | 71.0 |
|  | Errors | **3** | 124 | n/a | 70 | 6 | 4 | 4 | 90 |
|  | Errors-L | 5 | 14 | n/a | 3 | **2** | 39 | 7 | 53 |
|  | N50Corr (kb) | 70.1 | 8.5 | n/a | 36.2 | 12.8 | 147.2 | **215.4** | 60.0 |
|  | GenFrac (%) | 99.0 | 96.2 | n/a | 98.4 | 99.1 | 99.2 | **99.4** | 99.0 |
|  | Unaligned | **0** | 5 | n/a | 1 | 4 | 1 | 20 | 1 |
|  | Duplication | **1.0** | **1.0** | n/a | 1.1 | **1.0** | **1.0** | **1.0** | **1.0** |

**Supplementary Table S6**. Assemblies of *V. cholerae* HiSeq data (reference genome size: 4,033,464bp, number of proteins: 3,834)

|  |  | ABySS | CABOG | MIRA | MaSuRCA | SGA | SOAPdenovo | SPAdes | Velvet |
| --- | --- | --- | --- | --- | --- | --- | --- | --- | --- |
| Contigs | Num | 206 | 127 | 728 | **105** | 484 | 139 | 205 | 261 |
|  | N50 (kb) | 94.5 | 57.9 | 92.0 | **241.6** | 23.4 | 125.9 | 77.1 | 40.1 |
|  | Errors | 7 | 33 | 89 | 12 | **5** | 26 | 7 | 9 |
|  | Errors-L | 18 | 12 | 15 | 5 | **0** | 50 | 4 | 9 |
|  | N50Corr (kb) | 93.0 | 48.8 | 87.1 | **236.4** | 23.4 | 106.5 | 77.1 | 39.5 |
|  | GenFrac (%) | 99.6 | 96.6 | **99.7** | 99.4 | 99.3 | 99.5 | 99.6 | 99.4 |
|  | Unaligned | **0** | **0** | 10 | **0** | 1 | 5 | 8 | 1 |
|  | Duplication | **1.0** | **1.0** | **1.0** | **1.0** | **1.0** | **1.0** | **1.0** | **1.0** |
|  | Proteins | 3488 | 3318 | **3547** | 3529 | 3405 | 3512 | 3541 | 3451 |
| Scaffolds | Num | 102 | 108 | n/a | 88 | 331 | **75** | 106 | 85 |
|  | N50 (kb) | 217.6 | 67.0 | n/a | **246.5** | 23.4 | 181.1 | 98.3 | 172.5 |
|  | Errors | 18 | 34 | n/a | 11 | **5** | 26 | 27 | 13 |
|  | Errors-L | 70 | 24 | n/a | 8 | **0** | 76 | 19 | 132 |
|  | N50Corr (kb) | 157.1 | 53.2 | n/a | **236.4** | 23.4 | 168.1 | 94.8 | 171.5 |
|  | GenFrac (%) | 99.5 | 96.6 | n/a | 99.3 | 96.1 | 99.0 | **99.6** | 98.9 |
|  | Unaligned | **0** | **0** | n/a | **0** | 1 | 1 | 1 | 1 |
|  | Duplication | **1.0** | **1.0** | n/a | **1.0** | **1.0** | **1.0** | **1.0** | **1.0** |

**Supplementary Table S7**. Assemblies of *V. cholerae* MiSeq data (reference genome size: 4,967,469bp, number of proteins: 3,834)

|  |  | ABySS | CABOG | MIRA | MaSuRCA | SGA | SOAPdenovo | SPAdes | Velvet |
| --- | --- | --- | --- | --- | --- | --- | --- | --- | --- |
| Contigs | Num | 267 | 241 | 430 | **173** | 1721 | 244 | 1475 | 201 |
|  | N50 (kb) | 60.5 | 32.8 | 112.9 | 76.1 | 27.3 | 71.4 | **262.2** | 92.0 |
|  | Errors | **3** | 22 | 148 | 23 | 109 | 21 | 7 | 12 |
|  | Errors-L | **0** | 7 | 17 | 5 | 5 | 48 | 6 | 7 |
|  | N50Corr (kb) | 60.3 | 32.5 | 108.7 | 71.6 | 27.3 | 65.5 | **246.6** | 67.1 |
|  | GenFrac (%) | 99.3 | 97.8 | **99.6** | 98.3 | **99.6** | 99.3 | **99.6** | 99.5 |
|  | Unaligned | **0** | 1 | 20 | **0** | 6 | 4 | 1336 | 1 |
|  | Duplication | **1.0** | **1.0** | **1.0** | **1.0** | 1.2 | **1.0** | **1.0** | **1.0** |
|  | Proteins | 3512 | 3411 | 3557 | 3541 | 3446 | 3488 | **3564** | 3508 |
| Scaffolds | Num | 196 | 241 | n/a | 163 | 309 | 165 | **145** | 138 |
|  | N50 (kb) | 60.5 | 32.8 | n/a | 76.1 | 27.3 | 91.9 | **262.2** | 110.0 |
|  | Errors | **3** | 22 | n/a | 23 | 4 | 24 | 7 | 17 |
|  | Errors-L | **0** | 7 | n/a | 5 | 1 | 80 | 6 | 23 |
|  | N50Corr (kb) | 60.3 | 32.5 | n/a | 71.6 | 27.3 | 89.8 | **246.6** | 92.0 |
|  | GenFrac (%) | 98.5 | 97.8 | n/a | 98.3 | 96.4 | 98.7 | **99.6** | 99.2 |
|  | Unaligned | **0** | 1 | n/a | **0** | **0** | 1 | 57 | 1 |
|  | Duplication | **1.0** | **1.0** | n/a | **1.0** | **1.0** | **1.0** | **1.0** | **1.0** |

**Supplementary Table S8**. Assemblies of *A. hydrophila* HiSeq data (reference genome size: 4,744,448bp, number of proteins: 4,121)

|  |  | ABySS | CABOG | MIRA | MaSuRCA | SGA | SOAPdenovo | SPAdes | Velvet |
| --- | --- | --- | --- | --- | --- | --- | --- | --- | --- |
| Contigs | Num | 75 | 105 | 1048 | **32** | 201 | 61 | 312 | 65 |
|  | N50 (kb) | 237.5 | 278.4 | 246.2 | **828.6** | 68.8 | 243.9 | 379.7 | 184.4 |
|  | Proteins | 2900 | 2224 | 2906 | 2900 | 2870 | 2904 | **2906** | 2905 |
| Scaffolds | Num | 45 | 37 | n/a | **26** | 138 | 32 | 40 | 30 |
|  | N50 (kb) | 268.4 | 278.4 | n/a | **862.3** | 68.8 | 334.4 | 491.2 | 336.0 |

**Supplementary Table S9**. Assemblies of *B. cereus* HiSeq data (reference genome size: 5,432,652bp, number of proteins: 5,843)

|  |  | ABySS | CABOG | MIRA | MaSuRCA | SGA | SOAPdenovo | SPAdes | Velvet |
| --- | --- | --- | --- | --- | --- | --- | --- | --- | --- |
| Contigs | Num | 472 | **164** | 676 | 250 | 961 | 410 | 1041 | 376 |
|  | N50 (kb) | 48.3 | 61.6 | 47.4 | **103.6** | 23.4 | 57.9 | 97.2 | 38.9 |
|  | Proteins | 3337 | 2402 | 3101 | 3272 | 3316 | 3335 | **3346** | 3062 |
| Scaffolds | Num | 341 | **150** | n/a | 206 | 283 | 271 | 249 | 253 |
|  | N50 (kb) | 51.3 | 69.4 | n/a | **119.4** | 1.9 | 60.3 | 97.4 | 41.4 |

**Supplementary Table S10**. Assemblies of *B. fragilis* HiSeq data (reference genome size: 5,373,121bp, number of proteins: 4,290)

|  |  | ABySS | CABOG | MIRA | MaSuRCA | SGA | SOAPdenovo | SPAdes | Velvet |
| --- | --- | --- | --- | --- | --- | --- | --- | --- | --- |
| Contigs | Num | 158 | 137 | 400 | **119** | 487 | 246 | 146 | 213 |
|  | N50 (kb) | 146.2 | 94.2 | 134.3 | **158.7** | 41.2 | 116.1 | 157.7 | 125.2 |
|  | Proteins | 3154 | 2093 | 3155 | 3151 | 3112 | 3149 | **3156** | 3150 |
| Scaffolds | Num | 135 | 129 | n/a | **104** | 224 | 137 | 106 | 124 |
|  | N50 (kb) | 148.7 | 94.2 | n/a | **158.7** | 50.0 | 130.1 | 157.7 | 147.5 |

**Supplementary Table S11**. Assemblies of *S. aureus* HiSeq data (reference genome size: 2,903,081bp, number of proteins: 2,693)

|  |  | ABySS | CABOG | MIRA | MaSuRCA | SGA | SOAPdenovo | SPAdes | Velvet |
| --- | --- | --- | --- | --- | --- | --- | --- | --- | --- |
| Contigs | Num | 103 | 56 | 207 | **52** | 259 | 70 | 68 | 70 |
|  | N50 (kb) | 73.9 | 102.8 | 132.4 | **221.8** | 38.1 | 146.3 | 187.1 | 122.5 |
|  | Proteins | 2219 | 1797 | 2217 | 2205 | 2205 | 2220 | **2223** | 2222 |
| Scaffolds | Num | 83 | 53 | n/a | **38** | 97 | 50 | 44 | 52 |
|  | N50 (kb) | 80.1 | 153.4 | n/a | **221.9** | 51.1 | 146.3 | 187.1 | 146.3 |

**Supplementary Table S12**. Assemblies of *X. axonopodis* HiSeq data (reference genome size: 4,967,469bp, number of proteins: 4,178)

|  |  | ABySS | CABOG | MIRA | MaSuRCA | SGA | SOAPdenovo | SPAdes | Velvet |
| --- | --- | --- | --- | --- | --- | --- | --- | --- | --- |
| contigs | Num | 182 | **99** | 2750 | 155 | 313 | 202 | 191 | 214 |
|  | N50 (kb) | 89.9 | 105.8 | 105.6 | **117.9** | 47.8 | 74.2 | 117.5 | 83.0 |
|  | Proteins | 2668 | 2519 | 2676 | 2668 | 2631 | 2656 | **2681** | 2672 |
| scaffolds | Num | 136 | **89** | n/a | 136 | 204 | 138 | 153 | 139 |
|  | N50 (kb) | 94.2 | 105.8 | n/a | **117.9** | 47.8 | 91.2 | 117.5 | 105.3 |

***Determining whether a reference genome was close enough to a sequenced genome***

For several of the sequenced genomes, the finished reference genome from the same species represented a different strain, which made it more difficult to evaluate the accuracy of the assembled genomes. To determine whether an existing finished genome was close enough to the sequenced genomes used in GAGE-B, we mapped reads to the closest finished genome. We used Bowtie2 with default parameters and a generous range for the library insert size. The bowtie2 command was:

bowtie2 –I min –X max -1 reads1.fastq -2 reads2.fastq –S outputFile

with

min=80 max=280 for *A. hydrophila* HiSeq data, *B. cereus* HiSeq data, *B. fragilis* HiSeq data, *S. aureus* HiSeq data

min=95 max=345 for *R. sphaeroides* HiSeq data

min=160 max=510 for *M. abscessus* HiSeq data, *M. abscessus* MiSeq data, *V. cholerae* HiSeq data, *V. cholera* MiSeq data

min=160 max=510 for *R. sphaeroides* MiSeq data

min=200 max=600 for *X. axonopodis* HiSeq data

min=300 max=900 for *B. cereus* MiSeq data

Next, we looked what percent of reference genome was covered by this mapping with over 5x coverage, shown in Table S13.

Supplementary Table S13. Coverage of reference genomes based on mapping raw reads from each sequence data set with Bowtie2.

|  | Reference genome size (bp) | Number of bases covered (bp) | % covered |
| --- | --- | --- | --- |
| A. hydrophila HiSeq | 4,744,448 | 4,064,025 | 85.65 |
| B. cereus HiSeq | 5,432,652 | 3,138,766 | 57.78 |
| B. cereus MiSeq | 5,432,652 | 5,431,532 | 99.98 |
| B. fragilis | 5,373,121 | 4,367,917 | 81.29 |
| M. abscessus HiSeq | 5,090,401 | 5,060,999 | 99.42 |
| M. abscessus MiSeq | 5,090,401 | 5,021,173 | 98.64 |
| R. sphaeroides HiSeq | 4,565,960 | 4,564,190 | 99.96 |
| R. sphaeroides MiSeq | 4,565,960 | 4,560,486 | 99.88 |
| S. aureus HiSeq | 2,903,081 | 2,681,403 | 92.36 |
| V. cholera HiSeq | 4,033,464 | 4,009,169 | 99.40 |
| V. cholerae MiSeq | 4,033,464 | 3,879,892 | 96.19 |
| X. axonopodis HiSeq | 4,967,469 | 4,225,982 | 85.07 |

If at least 90% of the reference genome was covered by reads, we considered the reference genome to be similar enough to the sequenced genome to compute corrected N50 contig sizes; otherwise we only report the uncorrected N50 statistics.

***Data cleaning***

We used fastq-mcf utility from *ea-utils* package revision 396 with the following parameters to remove adapter sequences from reads and perform q10 quality trimming:

fastq-mcf –q 10 –t 0.01 adapter.fa reads_1.fastq reads_2.fastq

where adapter.fa file contained the adapter sequences:

>adapter_5p
AGATCGGAAGAGCACACGTCTGAACTCCAGTCAC
>adapter_3p
AGATCGGAAGAGCGTCGTGTAGGGAAAGAGTGTA

***Data used for the best genome assembly***

Each assembler was run on both raw and clean datasets, and the best assembly was reported. The datasets that generated the best assembly for each assembler are shown in Suppl. Table 14.

Supplementary Table S14. For each assembler and for each genome data set, we provided both the raw data and the “cleaned” data (see main text), and we chose the better assembly in each case. Shown is the data set chosen for each of the 12x8 combinations.

|  | ABySS | CABOG | MIRA | MaSuRCA | SGA | SOAPdenovo | SPAdes | Velvet |
| --- | --- | --- | --- | --- | --- | --- | --- | --- |
| *A. hydrophila* | raw | clean | raw | clean | clean | raw | clean | raw |
| *B. cereus -*HiSeq | raw | raw | raw | raw | raw | raw | clean | clean |
| *B. cereus -*MiSeq | raw | clean | raw | raw | raw | raw | clean | raw |
| *B. fragilis* | raw | clean | clean | clean | clean | raw | clean | raw |
| *M. abscessus -HiSeq* | clean | clean | raw | raw | clean | clean | clean | clean |
| *M. abscessus -MiSeq* | clean | clean | clean | clean | clean | raw | clean | raw |
| *R.sphaeroides -* HiSeq | raw | raw | raw | raw | raw | raw | clean | raw |
| *R.sphaeroides -* MiSeq | raw | raw | raw | raw | clean | clean | clean | clean |
| *S. aureus* | raw | raw | clean | raw | clean | raw | clean | clean |
| *V. cholerae -*HiSeq | clean | clean | raw | raw | clean | clean | clean | clean |
| *V. cholerae -*MiSeq | clean | raw | clean | clean | clean | raw | clean | clean |
| *X. axonopodis* | raw | clean | clean | raw | raw | raw | clean | raw |

***Recipes for genome assemblies***

This section lists the commands and parameters that generated the best assembly for each assembler.

To run **ABySS**, we used the following command:

abyss-pe k=K l=1 n=5 s=100 name=asm lib=’reads’ reads=reads.fastq aligner=bowtie

with the following values for the kmer parameter K:

K=31 for *R. sphaeroides* HiSeq data

K=49 for *B. cereus* MiSeq data, *R. sphaeroides* MiSeq data *X. axonipodis* HiSeq data

K=51 for *V. cholerae* HiSeq data

K=53 for *M. abscessus* HiSeq data

K=58 for *M. abscessus* MiSeq data

K=65 for *A. hydrophila* HiSeq data*, B. cereus* HiSeq data, *V. cholerae* MiSeq data, *S. aureus* HiSeq data

K=83 for *B. fragilis* HiSeq data

To run **CABOG**, we used the following commands:

fastqToCA -insertsize M S -libraryname reads -mates reads1.fastq,reads2.fastq > reads.frg

runCA –d . –p asm –s config reads.frg>&runCA.log

with config file specifying

unitiger = bog

and the corresponding mean M and standard deviation S of the library’s insert size:

M=180 and S=20 for *B. cereus* HiSeq data, *B. fragilis* HiSeq data, *A. hydrophila* HiSeq data, *S. aureus* HiSeq data

M=220 and S=25 for *R. sphaeroides* HiSeq data

M=335 and S=35 for *M. abscessus* HiSeq data, *M. abscessus* MiSeq data, *V. cholerae* HiSeq data, *V. cholerae* MiSeq data

M=400 and S=40 for *X. axonopodis* HiSeq data

M=540 and S=60 for *R. sphaeroides* MiSeq data

M=600 and S=60 for *B. cereus* MiSeq data

To run **MIRA**, we used the following commands:

srrname=SRRxxxxxx

numreads=xxxxxxx

strainname=”NA”

numlines=$((4*${numreads}))

cat reads1.fastq | head -${numlines} | sed -e ‘s/SRR[0-9.]*/&\/1/’ >${strainname}-${numreads} in.solexa.fastsq

cat reads2.fastq | head -${numlines} | sed -e ‘s/SRR[0-9.]*/&\/1/’ >${strainname}-${numreads}_in.solexa.fastsq

grep “@SRR” ${strainname}-${numreads}_in.solexa.fastq | cut –f 1 –d ‘ ‘ | sed -e ‘s/@//’ -e “s/$/ ${strainname}/” >> ${strainname}-${numreads}_straindata_in.txt

ln –s NA-numreads_in.solexa.fastq mira_in.solexa.fastq

ln –s NA-numreads_straindata_in.txt mira_straindata_in.txt

mira –fastq –job=denovo,genome,accurate,solexa SOLEXA_SETTINGS –GE:tismin=MIN:tismax=MAX –LR:file_type=fastq –AS:mrpc=5>&log_assembly.txt

with srrname and numreads containing the correct values for each run, and MIN and MAX having the following values:

MIN=90 and MAX=270 for *A. hydrophila HiSeq* data, *B. cereus* HiSeq data, *B. fragilis* HiSeq data, *S. aureus* HiSeq data

MIN=110 and MAX=330 for *R. sphaeroides* HiSeq data

MIN=167 and MAX=502 for *M. abscessus* HiSeq data, *M. abscessus* MiSeq data, *V. cholerae* HiSeq data, *V. cholerae* MiSeq data

MIN=200 and MAX=600 for *X. axonopodis* HiSeq data

MIN=270 and MAX=810 for *R. sphaeroides* MiSeq data

MIN=300 and MAX=900 for *B. cereus* MiSeq data

To run **MaSuRCA**, we used the following commands:

runSRCA.pl config

./assemble

where config file contains the following information (note that these runs used an earlier version of MaSuRCA that was abbreviated MSR-CA):

PATHS

JELLYFISH_PATH=/full/path/to/MSR-CA/bin

SR_PATH=/full/path/to/MSR-CA/bin

CA_PATH=/full/path/to/Cabog_installation/bin

END

DATA

PE= p1 M S reads1.fastq reads2.fastq

END

PARAMETERS

GRAPH_KMER_SIZE=K

NUM_THREADS=t

JF_SIZE=2000000000

END

with M and S set to correct mean and standard deviation values for a particular data set (see the values for M and S in the description of Cabog assembler), and the following values of kmer K were used:

K=49 for *B. cereus* HiSeq data

K=55 for *R. sphaeroides* HiSeq data

K=63 for *R. sphaeroides* MiSeq data

K=79 for *S. aureus* HiSeq data

K=89 for *A. hydrophila* HiSeq data, *B. fragilis* HiSeq data, *M. abscessus* HiSeq data, *V. cholerae* HiSeq data, *X. axonopodis* HiSeq data

K=99 for *M. abscessus* MiSeq data, *V. cholerae* HiSeq data

K=101 for *B. cereus* MiSeq data

To run **SGA**, we used the following script:

ln –s reads1.fastq frag1

ln –s reads2.fastq frag2

#!/bin/bash

K=kmer_value

CPU=8

MIN_OVERLAP=min_overlap

ASSEMBLE_OVERLAP=assemble_overlap

MIN_PAIRS=5

sga preprocess --pe-mode 1 –o reads.pp.fastq frag1 frag2

sga index --algorithm=ropebwt –t $CPU reads.pp.fastq

sga correct –k $K –t $CPU –o reads.ec.fastq reads.pp.fastq

sga index --algorithm=ropebwt –t $CPU reads.ec.fastq

sga filter –t $CPU reads.ec.fastq

sga overlap –m $MIN_OVERLAP –t $CPU reads.ec.filter.pass.fa

sga assemble –o primary reads.ec.filter.pass.asqg.gz

ln –s primary-contigs.fa.ctg.fasta

bwa index ctg.fasta

bwa aln –t $CPU ctg.fasta frag1 > frag1.sai

bwa aln –t $CPU ctg.fasta frag2 > frag2.sai

bwa sampe ctg.fasta frag1.sai frag2.sai frag1 frag2 > frag.sam

samtools view –Sb frag.sam > libPE.bam

sga-bam2de.pl –n $MIN_PAIRS --prefix libPE libPE.bam

sga-astat.py libPE.bam > libPE.astat

sga scaffold –m 200 –a libPE.astat –o scf --pe libPE.de ctg.fasta

sga scaffold2fasta –a primary-graph.asqg.gz –o scf.fasta scf

with the following values used for kmer_value (K), min_overlap (M), and assemble_overlap (A):

K=23, M=85, A= 111 for *R. sphaeroides* MiSeq data

K=41, M=45, A= 45 for *R. sphaeroides* HiSeq data

K=55, M=45, A= 45 for *A. hydrophila* HiSeq data

K=65, M=45, A= 45 for *B.cereus* HiSeq data, *B. fragilis* HiSeq data, *M. abscessus* HiSeq data, *V. cholerae* HiSeq data, *X. axonopodis* HiSeq data

K=73, M=45, A= 45 for *S. aureus* HiSeq data

K=65, M=85, A= 111 for *B.cereus* MiSeq data, *M. abscessus* MiSeq data, *V. cholerae* MiSeq data

To run **SOAPdenovo2**, we configured the software to allow using 127mer, and used the following command:

SOAPdenovo2 all –K kmer_value –F –R –E –w –u –s config –o asm –p 8>> SOAPdenovo.log

GapCloser –b config –a asm.scafSeq –o asm.new.scafSeq –t 8 >> SOAPdenovo.log

with config file containing the following information:

[LIB]

avg_ins=mean

reverse_seq=0

asm_flags=3

rank=1

q1=reads1.fastq

q2=reads2.fastq

with corresponding mean value for insert size (see cabog values for M), and with kmer_value:

K=47 *M. abscessus* MiSeq data

K=49 *M. abscessus* HiSeq data, *V. cholerae* MiSeq data

K=51 *V. cholerae* HiSeq data

K=55 for *B. cereus* MiSeq data *, R. sphaeroides* HiSeq data

K=65 for *B. cereus* HiSeq data

K=71 for *S. aureus* HiSeq data

K=79 for *A. hydrophila* HiSeq data, *B. fragilis* HiSeq data, *R. sphaeroides* MiSeq data, *X. axonopodis* HiSeq data

NOTE: For SOAPdenovo2, we also ran its GapCloser tool after completing the assembly, and extracted contigs from the gap-closed scaffolds in the same manner as for Velvet. GapCloser often produces significantly larger contigs and scaffolds.

To run **SPAdes** , we used the following commands:

spades.py –t 2 –k K1,K2,K3 -1 reads1.fastq –s reads2.fastq –o output

with the following values for kmer values K1,K2,K3:

21,33,55 for *R. sphaeroides* HiSeq data

31,43,65 for *R. sphaeroides* MiSeq data

41,53,75 for *B. cereus* HiSeq data

51,63,85 for *B. cereus* MiSeq data, *X. axonopodis* HiSeq data

61,73,95 for *A. hydrophila* HiSeq data, *B. fragilis* HiSeq data, *S. aureus* HiSeq data

33,55,65,75,85,99 for *M. abscessus* HiSeq data, *M. abscessus* MiSeq data, *V. cholerae* HiSeq data, *V. cholerae* MiSeq data

To run **Velvet**, we used the following commands:

shuffleSequences_fastq.pl reads1.fastq reads2.fastq inputReads.fastq

velveth . K –fastq –shortPaired inputReads.fastq

velvetg . –exp_cov auto –ins_length M –ins_length_sd S –scaffolding yes

with the corresponding mean M and standard deviation S (see values for M and S in cabog description), and the following values for kmer K:

K=31 for *R. sphaeroides* MiSeq data

K=49 for *R. sphaeroides* HiSeq data, *M. abscessus* HiSeq data, *V. cholerae* HiSeq data

K=63 for *A. hydrophila* HiSeq data, *B. cereus* HiSeq data, *B. cereus* MiSeq data, *X. axonopodis* HiSeq data

K=73 for *B. fragilis* Hiseq data, *S. aureus* HiSeq data

K=97 for *M. abscessus* MiSeq data, *V. cholerae* MiSeq data

NOTE: Velvet provides either contigs or scaffolds but not both in the same run. We ran velvet to scaffolding, and then extracted contigs from scaffolds by extracting each subsequence that contains no “N”s.

***Nx statistics***

The N50 size is a weighted average length statistic, defined as the length of the smallest contig such that 50% of the genome is contained in contigs of that size or larger. More generally, the Nx size is the length of the smallest contig such that x% of genome is contained in contigs of size Nx or larger. In Figures S1 and S2, we present Nx statistics in a graph that displays the Nx contig length (in kilobases) on the vertical axis and the x-value on the horizontal axis. Figure S1 shows these values for *M. abscessus*, and Figure S2 for *R. sphaeroides*. For example, the N20 value for SPAdes in Figure S1 is 280Kb, meaning that 10% of the genome is contained in contigs that are 280Kb or longer.


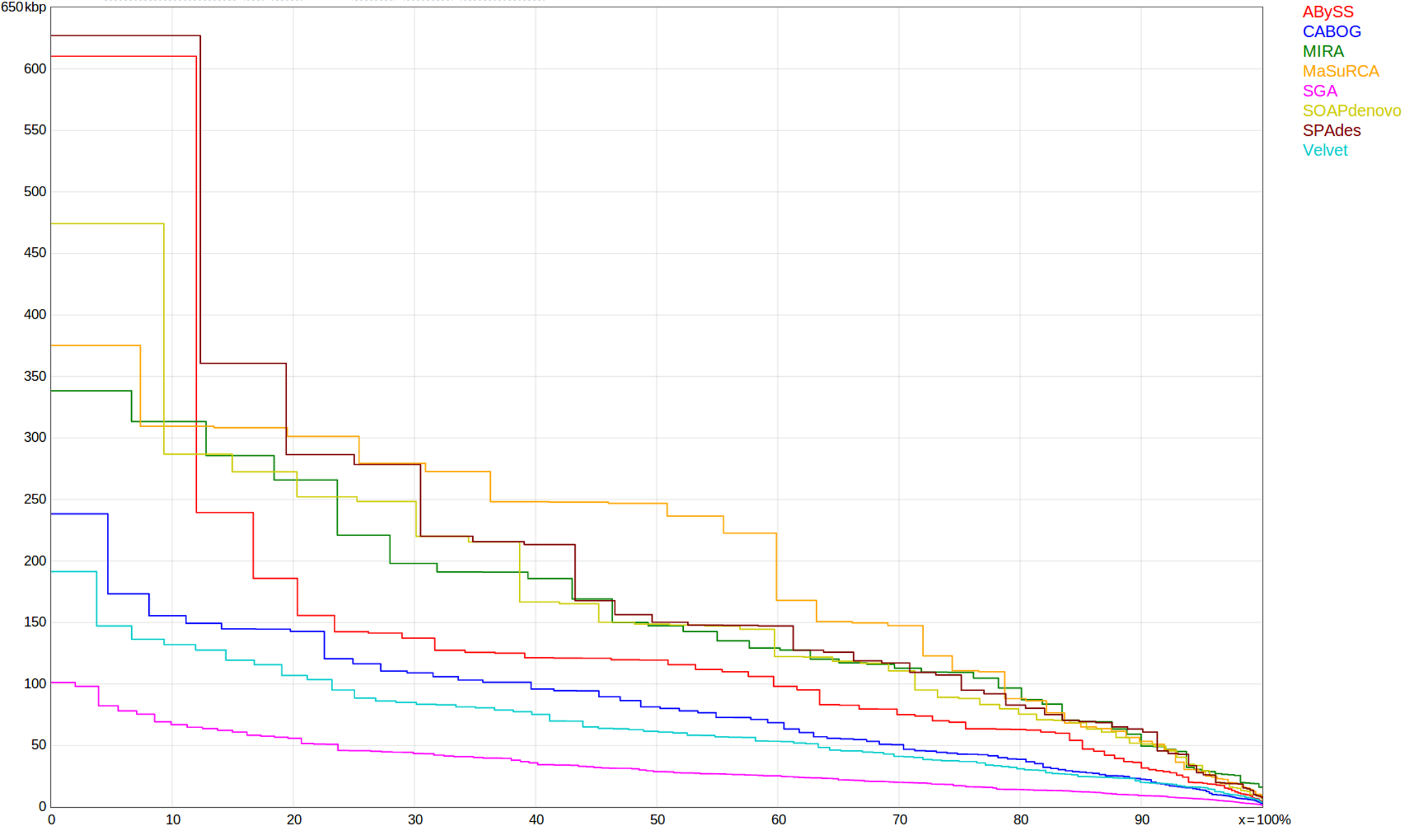


**Supplementary Figure S1.** Nx statistics for *M. abscessus* HiSeq assemblies.


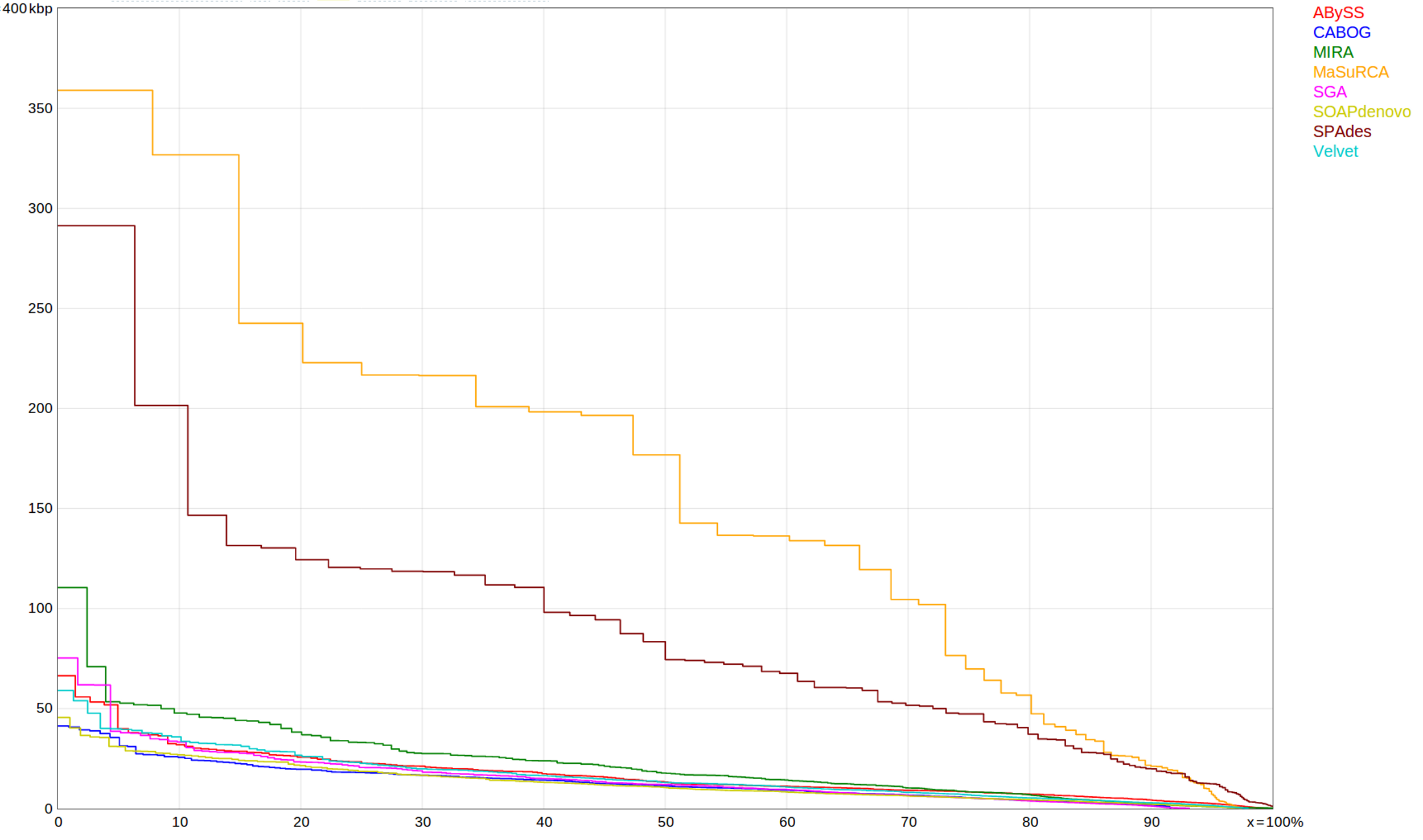


**Supplementary Figure S2.** Nx statistics for *R. sphaeroides* HiSeq assemblies.

***Determining whether proteins are contained in assembled contigs***

For each assembly, a database of contigs was created, and proteins annotated in the reference genomes were mapped against this database using tblastn with the following command:

makeblastdb -in <CTG_FILE> -dbtype nucl -out <DB> -input_type fasta

tblastn -num_threads 2 -comp_based_stats F -query <PROTEINS> -db <DB> -out report.bls

Next, for each protein query, the top hit and the top high-scoring segment pair (hsp) for which the hit length is equal to the protein length (i.e., the protein maps end-to-end to a contig) were chosen as the mapping of the protein to the contig. For these end-to-end matches, the protein was counted as contained in the assemblies’ contigs.

When the reference genome is the same strain as the sequenced genome, the identity level of proteins contained in contigs is usually over 95%. This is illustrated by the histogram for *R. sphaeroides* below, which shows the mapping of proteins to contigs from the SPAdes assembly of MiSeq data. The x-axis shows the percent identity of proteins aligned to the assembly. This same quantity varies much more when the reference genome is not as close to the sequenced strain, as shown in the histogram of alignments for the *B. cereus* HiSeq data, which represents mapping of proteins to contigs from the SPAdes assembly of that data.

**Supplementary Figure S3.** Mapping of proteins to the reference genome of the same strain (*R. sphaeroides*) and the reference genome of a related strain (*B. cereus*), showing the number of proteins mapping at different levels of identity.

***Combining assemblies***

Merging pairs of contig sets using minimus2 yielded sets of sequences whose total length often differed considerably from the true genome size, often being significantly larger (Table S15). Clustering the contigs using CD-HIT usually reduced the total length, but in most cases it still exceeded the target size (Table S16). For pairs of assemblies that yielded contig sets whose length was within 10% of the target size, we present performance statistics in Table S19. Combining MaSuRCA and CABOG marginally improved upon the N50 size of the best individual assembly (MaSuRCA), at the cost of a slightly higher number of errors. Similarly, the combination of the MIRA and Velvet assemblies slightly increased the N50 size, but again introduced more errors.

On the other hand, considering only contigs that were merged (ignoring unmerged contigs) created sequence sets that were too small (Table S17). Only combinations that include the SPAdes assembly yielded a result longer than the target size, but this was because the SPAdes assembly itself had a total length of almost 19 Mbp. If the clustering step was omitted the total length of the merged contigs was often either too long or too short (Table S18). For merged assemblies whose total length was within 10% of the target size, we present statistics in Table S20. The combinations of the MIRA and the MaSuRCA assemblies represent a substantial improvement in the N50 size, and the number of errors slightly decreased. However, only approximately 90% of the reference genome was covered in this case. We observed similar phenomena for other pairs too: the CABOG/ABySS combination exhibited a higher (corrected) N50 value than either the CABOG or the ABySS assembly but covered only ~90% of the reference genome. On the positive side, the ABySS/Velvet combination increased the (corrected) N50 value of the two individual assemblies significantly while covering more than 99% of the reference genome. More surprisingly, in the SPAdes/SGA combination SGA seems to act as a filter for the low coverage contigs that did not align to the reference genome (see discussion of the SPAdes assembly above). From the almost 40,000 unaligned contigs only 186 remain. At the same time, the (corrected) N50 value slightly increased compared to the two individual assemblies and the number of errors decreases, while covering 99.94% of the reference genome.

We observed more promising results when using GAA to merge two assemblies on the contig level. Table S21 shows that the total length of the assemblies produced by GAA is always close to the target size, with ABySS/MaSuRCA being the only notable exception. It should also be noted that GAA is more sensitive to the order of the input assemblies, because it is designed to improve the target assembly given a query assembly. Assemblies computed by SGA and SPAdes were excluded from this analysis, because their total length exceeded the target size significantly.

Finally, we investigated whether a combination of assemblies computed by MaSuRCA for different k-mer sizes can yield better results than selecting a single k-mer size (Table S22). We considered k-mer sizes of 49, 65, 69, and 101. Although the GAA combinations of the contigs had the correct total size, we observed no improvement compared to a single assembly using k=101.

**Supplementary Table S15.** Total length (Mbp) of the union of merged and unmerged contigs computed by minimus2 for pairs of assemblies of *B. cereus* from 250bp MiSeq reads.

|  | ABySS | CABOG | MIRA | MaSuRCA | SGA | SOAPdenovo | SPAdes | Velvet |
| --- | --- | --- | --- | --- | --- | --- | --- | --- |
| ABySS |  | 9.2 | 9.9 | 9.9 | 9.7 | 10.0 | 20.3 | 6.8 |
| CABOG | 9.2 |  | 9.0 | 9.8 | 9.0 | 9.0 | 19.2 | 8.2 |
| MIRA | 9.9 | 8.9 |  | 8.7 | 8.4 | 9.3 | 19.5 | 8.5 |
| MaSuRCA | 9.8 | 9.8 | 8.7 |  | 9.0 | 9.8 | 19.7 | 8.1 |
| SGA | 9.7 | 8.9 | 8.4 | 9.0 |  | 8.9 | 18.4 | 9.1 |
| SOAPdenovo | 9.9 | 8.9 | 9.3 | 9.8 | 8.9 |  | 17.7 | 7.4 |
| SPAdes | 20.3 | 19.2 | 19.5 | 19.7 | 18.4 | 17.7 |  | 18.0 |
| Velvet | 8.8 | 8.2 | 8.5 | 8.1 | 9.1 | 7.4 | 18.0 |  |

Supplementary Table S16. Total length (Mbp) of the union of merged and unmerged contigs computed by minimus2 with initial clustering using CD-HIT for pairs of assemblies of *B. cereus* from 250bp MiSeq reads.

|  | ABySS | CABOG | MIRA | MaSuRCA | SGA | SOAPdenovo | SPAdes | Velvet |
| --- | --- | --- | --- | --- | --- | --- | --- | --- |
| ABySS |  | 8.1 | 8.4 | 8.9 | 6.4 | 9.1 | 18.0 | 6.2 |
| CABOG | 8.2 |  | 6.2 | 5.9 | 5.5 | 7.5 | 17.3 | 5.8 |
| MIRA | 8.4 | 6.2 |  | 6.4 | 5.5 | 7.2 | 17.0 | 6.0 |
| MaSuRCA | 8.9 | 5.9 | 6.4 |  | 5.5 | 8.1 | 17.2 | 5.8 |
| SGA | 6.4 | 5.5 | 5.5 | 5.5 |  | 6.0 | 16.0 | 6.3 |
| SOAPdenovo | 9.1 | 7.5 | 7.2 | 8.1 | 6.0 |  | 16.8 | 5.8 |
| SPAdes | 18.1 | 17.3 | 17.0 | 17.2 | 16.0 | 16.8 |  | 16.1 |
| Velvet | 6.2 | 5.8 | 6.0 | 5.7 | 6.3 | 5.8 | 16.1 |  |

Supplementary Table S17. Total length (Mbp) of merged contigs computed by minimus2 with initial clustering using CD-HIT for pairs of assemblies of *B. cereus* from 250bp MiSeq reads.

|  | ABySS | CABOG | MIRA | MaSuRCA | SGA | SOAPdenovo | SPAdes | Velvet |
| --- | --- | --- | --- | --- | --- | --- | --- | --- |
| ABySS |  | 4.4 | 4.4 | 4.4 | 3.4 | 4.5 | 8.1 | 3.7 |
| CABOG | 4.2 |  | 2.2 | 1.3 | 2.5 | 2.4 | 6.7 | 1.1 |
| MIRA | 4.1 | 2.0 |  | 1.9 | 3.0 | 2.5 | 7.0 | 1.3 |
| MaSuRCA | 4.4 | 1.3 | 1.9 |  | 3.7 | 2.9 | 6.4 | 1.7 |
| SGA | 3.4 | 2.5 | 3.0 | 3.7 |  | 3.1 | 5.7 | 2.5 |
| SOAPdenovo | 5.1 | 2.4 | 2.5 | 2.9 | 3.1 |  | 6.8 | 2.6 |
| SPAdes | 8.1 | 7.1 | 6.9 | 6.8 | 5.6 | 6.9 |  | 5.7 |
| Velvet | 3.6 | 1.1 | 1.4 | 1.7 | 2.6 | 2.6 | 5.8 |  |

Supplementary Table S18. Total length (Mbp) of merged contigs computed by minimus2 (no initial clustering) for pairs of assemblies of *B. cereus* from 250bp MiSeq reads.

| Assembly | ABySS | CABOG | MIRA | MaSuRCA | SGA | SOAPdenovo | SPAdes | Velvet |
| --- | --- | --- | --- | --- | --- | --- | --- | --- |
| ABySS |  | 6.0 | 7.0 | 6.5 | 6.3 | 6.5 | 9.1 | 5.8 |
| CABOG | 6.0 |  | 4.0 | 2.6 | 5.4 | 4.2 | 7.1 | 4.2 |
| MIRA | 6.8 | 4.2 |  | 5.7 | 5.4 | 5.2 | 7.8 | 4.5 |
| MaSuRCA | 6.7 | 2.5 | 5.3 |  | 5.5 | 4.8 | 7.9 | 4.3 |
| SGA | 6.4 | 5.5 | 5.5 | 5.7 |  | 5.6 | 7.6 | 3.6 |
| SOAPdenovo | 6.0 | 4.2 | 5.3 | 4.8 | 5.4 |  | 7.2 | 4.8 |
| SPAdes | 9.4 | 7.8 | 7.4 | 9.1 | 6.0 | 7.9 |  | 6.4 |
| Velvet | 4.2 | 4.2 | 4.5 | 4.3 | 3.7 | 4.7 | 7.5 |  |

Supplementary Table S19. Combined assemblies (merged and unmerged contigs) of *B. cereus* MiSeq data computed using minimus2 with initial clustering by CD-HIT. Only combined contig sets with total length within 10% of target size are depicted.

|  | Num | N50 (kb) | Errors | Errors-L | N50Corr (kb) | GenFrac (%) | Unaligned | Dup |
| --- | --- | --- | --- | --- | --- | --- | --- | --- |
| CABOG/MaSuRCA | 61 | 276.4 | 20 | 13 | 255.2 | 99.98 | 0 | 1.1 |
| CABOG/SGA | 262 | 155.4 | 30 | 10 | 150.5 | 99.98 | 2 | 1.1 |
| CABOG/Velvet | 97 | 155.4 | 14 | 16 | 150.5 | 99.94 | 0 | 1.1 |
| MIRA/SGA | 192 | 126.0 | 26 | 19 | 102.7 | 99.99 | 8 | 1.0 |
| MIRA/Velvet | 127 | 124.4 | 15 | 25 | 104.1 | 99.98 | 6 | 1.1 |
| MaSuRCA/CABOG | 61 | 276.4 | 20 | 13 | 255.2 | 99.98 | 0 | 1.1 |
| MaSuRCA/SGA | 205 | 246.7 | 27 | 13 | 246.7 | 99.98 | 3 | 1.1 |
| MaSuRCA_Velvet | 71 | 255.5 | 17 | 19 | 246.7 | 99.96 | 0 | 1.1 |
| SGA/CABOG | 262 | 155.4 | 30 | 10 | 150.5 | 99.98 | 1 | 1.1 |
| SGA/MIRA | 191 | 126.0 | 26 | 19 | 102.7 | 99.99 | 8 | 1.0 |
| SGA/MaSuRCA | 204 | 246.7 | 27 | 13 | 246.7 | 99.98 | 3 | 1.1 |
| SOAPdenovo/Velvet | 92 | 246.3 | 6 | 32 | 246.3 | 99.87 | 0 | 1.1 |
| Velvet/CABOG | 97 | 155.4 | 14 | 16 | 150.5 | 99.94 | 0 | 1.1 |
| Velvet/MaSuRCA | 71 | 255.5 | 17 | 19 | 246.7 | 99.96 | 0 | 1.1 |
| Velvet/SOAPdenovo | 92 | 246.3 | 6 | 32 | 246.3 | 99.87 | 0 | 1.1 |

Supplementary Table S20. Combined assemblies (merged contigs only) of *B. cereus* MiSeq data computed using minimus2 (no initial clustering). Only combined contig sets with total length within 10% of target size are depicted.

|  | Num | N50 (kb) | Errors | Errors-L | N50Corr (kb) | GenFrac (%) | Unaligned | Dup |
| --- | --- | --- | --- | --- | --- | --- | --- | --- |
| ABySS/Velvet | 92 | 211.4 | 9 | 39 | 170.5 | 99.02 | 0 | 1.1 |
| CABOG/ABySS | 51 | 255.2 | 11 | 20 | 211.9 | 90.98 | 0 | 1.2 |
| CABOG/SGA | 179 | 155.4 | 12 | 7 | 150.5 | 97.18 | 2 | 1.1 |
| MIRA/MaSuRCA | 36 | 338.2 | 6 | 15 | 338.2 | 90.15 | 0 | 1.2 |
| MIRA/SGA | 155 | 124.4 | 12 | 13 | 102.7 | 96.78 | 2 | 1.1 |
| MIRA/SOAPdenovo | 42 | 234.6 | 7 | 24 | 234.6 | 82.78 | 0 | 1.2 |
| MaSuRCA/MIRA | 34 | 338.2 | 5 | 15 | 338.2 | 88.33 | 0 | 1.1 |
| MaSuRCA/SGA | 160 | 246.7 | 12 | 10 | 246.7 | 99.95 | 1 | 1.1 |
| SGA/CABOG | 241 | 155.4 | 12 | 7 | 150.5 | 97.24 | 2 | 1.1 |
| SGA/MIRA | 218 | 124.4 | 14 | 13 | 102.7 | 96.78 | 3 | 1.1 |
| SGA/MaSuRCA | 222 | 246.7 | 14 | 10 | 246.7 | 99.95 | 2 | 1.1 |
| SGA/SOAPdenovo | 337 | 246.3 | 5 | 26 | 246.3 | 96.76 | 3 | 1.2 |
| SOAPdenovo/ABySS | 61 | 311.9 | 2 | 33 | 311.9 | 92.74 | 0 | 1.2 |
| SOAPdenovo/MIRA | 40 | 246.3 | 7 | 25 | 246.3 | 87.21 | 0 | 1.1 |
| SOAPdenovo/SGA | 318 | 246.3 | 5 | 26 | 246.3 | 96.39 | 3 | 1.1 |
| SPAdes/SGA | 504 | 116.5 | 10 | 12 | 116.5 | 99.94 | 186 | 1.1 |

Supplementary Table S21. Combined assemblies of *B. cereus* MiSeq data computed using GAA. Assemblies from SPAdes and SGA excluded.

|  | Num | Total length (Mbp) | N50 (kb) | Errors | Errors-L | N50Corr (kb) | GenFrac (%) | Unaligned | Dup |
| --- | --- | --- | --- | --- | --- | --- | --- | --- | --- |
| ABySS/CABOG | 115 | 5.41 | 130.6 | 4 | 28 | 130.6 | 99.54 | 2 | 1.0 |
| ABySS/MIRA | 115 | 5.40 | 130.6 | 3 | 29 | 130.6 | 99.61 | 2 | 1.0 |
| ABySS/MaSuRCA | 205 | 10.83 | 429.5 | 18 | 37 | 429.2 | 99.96 | 2 | 2.0 |
| ABySS/SOAPdenovo | 115 | 5.40 | 130.6 | 3 | 27 | 130.6 | 99.48 | 2 | 1.0 |
| ABySS/Velvet | 115 | 5.40 | 130.6 | 4 | 27 | 130.6 | 99.66 | 2 | 1.0 |
| CABOG/ABySS | 40 | 5.43 | 288.3 | 11 | 12 | 288.1 | 99.70 | 0 | 1.0 |
| CABOG/MIRA | 73 | 5.43 | 169.1 | 8 | 7 | 161.6 | 99.92 | 0 | 1.0 |
| CABOG/MaSuRCA | 53 | 5.43 | 263.2 | 10 | 7 | 247.3 | 99.98 | 0 | 1.0 |
| CABOG/SOAPdenovo | 47 | 5.43 | 288.2 | 9 | 11 | 288.2 | 99.52 | 0 | 1.0 |
| CABOG/Velvet | 79 | 5.42 | 155.4 | 8 | 6 | 150.5 | 99.94 | 0 | 1.0 |
| MIRA/ABySS | 152 | 5.44 | 116.5 | 11 | 18 | 100.0 | 99.98 | 7 | 1.0 |
| MIRA/CABOG_gaa | 153 | 5.44 | 116.5 | 11 | 18 | 100.0 | 99.98 | 7 | 1.0 |
| MIRA/MaSuRCA | 152 | 5.43 | 116.5 | 11 | 18 | 100.0 | 99.98 | 7 | 1.0 |
| MIRA/SOAPdenovo | 152 | 5.42 | 116.5 | 11 | 18 | 100.0 | 99.98 | 7 | 1.0 |
| MIRA/Velvet | 153 | 5.42 | 116.5 | 11 | 18 | 100.0 | 99.99 | 7 | 1.0 |
| MaSuRCA/ABySS | 88 | 5.46 | 271.1 | 15 | 10 | 271.1 | 99.87 | 0 | 1.0 |
| MaSuRCA/CABOG | 90 | 5.50 | 256.9 | 19 | 10 | 247.3 | 99.95 | 0 | 1.0 |
| MaSuRCA/MIRA | 89 | 5.44 | 246.7 | 15 | 11 | 246.7 | 99.90 | 0 | 1.0 |
| MaSuRCA/SOAPdenovo | 90 | 5.44 | 246.7 | 15 | 10 | 246.7 | 99.85 | 0 | 1.0 |
| MaSuRCA/Velvet | 90 | 5.44 | 246.7 | 15 | 10 | 246.7 | 99.90 | 0 | 1.0 |
| SOAPdenovo/ABySS | 87 | 5.41 | 457.8 | 2 | 33 | 457.1 | 99.27 | 0 | 1.0 |
| SOAPdenovo/CABOG | 85 | 5.42 | 458.7 | 4 | 22 | 458.1 | 99.61 | 0 | 1.0 |
| SOAPdenovo/MIRA | 92 | 5.37 | 246.6 | 2 | 24 | 246.6 | 99.27 | 0 | 1.0 |
| SOAPdenovo/MaSuRCA | 96 | 5.39 | 457.5 | 3 | 20 | 456.9 | 99.60 | 0 | 1.0 |
| SOAPdenovo/Velvet | 98 | 5.36 | 246.4 | 2 | 25 | 246.4 | 99.82 | 0 | 1.0 |
| Velvet/ABySS | 138 | 5.38 | 121.9 | 6 | 22 | 121.9 | 99.62 | 0 | 1.0 |
| Velvet/CABOG | 156 | 5.40 | 89.0 | 5 | 16 | 89.0 | 99.70 | 0 | 1.0 |
| Velvet/MIRA | 174 | 5.37 | 72.8 | 7 | 15 | 72.8 | 99.71 | 0 | 1.0 |
| Velvet/MaSuRCA | 120 | 5.39 | 152.1 | 8 | 17 | 152.1 | 99.74 | 0 | 1.0 |
| Velvet/SOAPdenovo | 132 | 5.35 | 166.7 | 5 | 24 | 166.7 | 99.67 | 0 | 1.0 |

Supplementary Table S22. GAA combinations of assemblies of *B. cereus* MiSeq data computed by MaSuRCA for k-mer sizes 49, 65, 69, and 101 (left-most column).

|  | Num | Total length (Mbp) | N50 (kb) | Errors | Errors-L | N50Corr (kb) | GenFrac (%) | Unaligned | Dup |
| --- | --- | --- | --- | --- | --- | --- | --- | --- | --- |
| 101/49 | 90 | 5.46 | 246.7 | 15 | 10 | 246.7 | 99.85 | 0 | 1.0 |
| 101/65 | 90 | 5.45 | 246.7 | 15 | 10 | 246.7 | 99.87 | 0 | 1.0 |
| 101/69 | 89 | 5.44 | 246.7 | 15 | 10 | 246.7 | 99.87 | 0 | 1.0 |
| 49/101 | 83 | 5.51 | 236.1 | 12 | 16 | 212.4 | 99.88 | 0 | 1.0 |
| 49/65 | 80 | 5.46 | 236.1 | 12 | 16 | 212.4 | 99.77 | 0 | 1.0 |
| 49/69 | 80 | 5.45 | 236.1 | 11 | 16 | 212.4 | 99.84 | 0 | 1.0 |
| 65/101 | 93 | 5.47 | 217.4 | 14 | 18 | 212.4 | 99.93 | 0 | 1.0 |
| 65/49 | 90 | 5.53 | 242.2 | 13 | 18 | 212.4 | 99.81 | 0 | 1.0 |
| 65/69 | 91 | 5.47 | 217.4 | 12 | 19 | 212.4 | 99.88 | 0 | 1.0 |
| 69/101 | 92 | 5.45 | 212.1 | 13 | 17 | 212.1 | 99.82 | 0 | 1.0 |
| 69/49 | 91 | 5.45 | 212.1 | 11 | 15 | 212.1 | 99.80 | 0 | 1.0 |
| 69/65 | 92 | 5.45 | 212.1 | 11 | 16 | 212.1 | 99.80 | 0 | 1.0 |
